# Efficacy and mechanisms of an education outside the classroom intervention on pupils’ health and education: the MOVEOUT study protocol

**DOI:** 10.1186/s12889-023-16618-3

**Published:** 2023-09-19

**Authors:** Mads Bølling, Lærke Mygind, Peter Elsborg, Paulina S. Melby, Karen S. Barfod, Jan Christian Brønd, Charlotte Demant Klinker, Glen Nielsen, Peter Bentsen

**Affiliations:** 1 Center for Clinical Research and Prevention, Copenhagen University Hospital – Bispebjerg and Frederiksberg, the Capital Region of Denmark, Copenhagen, Denmark; 2 Health Promotion Research, Copenhagen University Hospital – Steno Diabetes Center Copenhagen, Herlev, Denmark; 3 Research Centre for Pedagogy and Bildung, Program on Outdoor Pedagogy, VIA University College, Aarhus, Denmark; 4 Department of Sports Science and Clinical Biomechanics, University of Southern Denmark, Odense, Denmark; 5 Department of Nutrition, Exercise and Sports, University of Copenhagen, Copenhagen, Denmark; 6 Department of Geoscience and Natural Resource Management, University of Copenhagen, Copenhagen, Denmark

**Keywords:** Learning outside the classroom, Movement integration, Outdoor learning, Outdoor teaching, School-based health promotion, Structural prevention

## Abstract

**Background:**

Education can create better opportunities for health, and vice versa. Using a so-called ‘add-in’ approach, school-based physical activity (PA) promotion and prevention of sedentary behaviours can increase pupils’ wellbeing and learning and, on the longer term, reduce the risk of non-communicable diseases. A PA ‘add-in’ approach involves integrating PA into teachers’ curricular obligations without being an extra burden as opposed to an ‘add-on’ approach which requires additional operational resources and include activities that do not explicitly contribute towards curricular targets making them less long-term acceptable in a school-based context.

Previous studies investigating education outside the classroom (EOtC) show mutual benefits for both health and education outcomes among children and adolescents. However, the evidence is of mixed quality and questionable certainty, which calls for further investigation. The aim of this study protocol is to describe and discuss the study design and methods to investigate the efficacy and mechanisms of EOtC as a vehicle for health and education. The study investigates the intervention developed and conducted in the TEACHOUT study with updated and strengthened design and measures.

**Methods:**

The efficacy of EOtC will be investigated in a cluster randomised waitlist design. Participants will be pupils in ~54 classes, grades 4-10 (ages 10-15 years) in ~30 Danish elementary schools. Fifteen schools will be randomised to the intervention: a two-day EOtC training course targeting teachers followed by the teachers implementing EOtC >5 hours weekly over the course of one school year. Pre- and post-measures of health (PA and wellbeing) and learning (school motivation and academic achievement) will be collected. Investigation of pedagogical and motivational mechanisms will be based on observations of EOtC.

**Discussion:**

The updated randomised controlled design will provide firmer evidence for the efficacy and mechanisms of EOtC and provide knowledge about how mutual benefits of health and education can be obtained.

**Trial registration:**

Registered with ClinicalTrials.gov (ID NCT05237674) [University of Copenhagen. MOVEOUT: a Cluster RCT of the Efficacy, Mechanisms, and Mediation of an Education Outside the Classroom Intervention on Adolescents’ Physical Activity, 2023], February 14, 2022. Most recently updated on November 23, 2022 (Version 2).

**Supplementary Information:**

The online version contains supplementary material available at 10.1186/s12889-023-16618-3.

## Background

Health and education are synergistic in multifarious ways. Good health supports wellbeing and the cognitive functions underlying learning over the life course [1]. Vice versa, poor health can interfere negatively with schooling, and informal learning in general, through recurrent absences or difficulties concentrating [2, 3]. Good opportunities for learning, on the other hand, provides the basis for participation and informed lifestyles choices. If either education or health is hampered in early life, this will have incremental impacts for both over the life course. Negative feedback loops in childhood tend to track into in adulthood where individuals with higher education generally have higher earnings (and in extension opportunities for healthier foods and living conditions), are more likely to be employed, and to take up positions with fewer risks and physical strains than individuals with lower levels of education [4]. The interconnectedness of health and education calls for coordinated solutions for which efficient and sustainable early-life interventions that can support both health and education in tandem are a necessity. Health and education synergies can be maximised through intersectoral actions, but these actions require a sensitivity towards the core goals, processes, and mandates of the settings through which the actions are implemented. In this protocol, we outline one such approach and describe the methods planned to investigate the efficacy and mechanism of an intervention.

Schools are considered key settings for promoting children and adolescents’ health and education, as they spend a large amount of their waking hours at school [5, 6], and because children and adolescents from all socioeconomic and cultural backgrounds can be reached [5]. However, school-based intervention requires an integration of the associated activities within the core goals, processes, and mandates of schools for successful and sustainable implementation [7]. This is particularly pertinent given the current education milieu in which schools and teachers face external top-down pressures to improve academic standards and improve wellbeing among pupils [8]. This means that there is a need for teaching activities and learning experiences that simultaneously improve pupils’ learning, wellbeing, and health.

Increased physical activity (PA) and decreased sedentary time reduces the risk of non-communicable diseases, such as cardiovascular diseases, diabetes, and morbidity over time [9]. Positive effects are also seen on wellbeing, cognitive functioning [10], and academic achievement during childhood [11]. European adolescents are far from reaching the PA guidelines, and activity levels decline with increasing age [12]. Furthermore, the decreasing levels of wellbeing and rise of mental health problems among adolescents has increased during the recent years [13]. Various attempts have been made to increase PA during school hours, e.g., through extracurricular activities such as active brain breaks or time slots allocated for movement activities [14, 15]. However, these approaches are often disconnected from school objectives, require additional manpower, or increasing workload for existing personnel and is not considered a primary objective by the teachers, which in many cases cause a low degree of implementation [16]. In contrast, ‘add-in’ approaches that integrate PA within primary pedagogical and didactical purposes have shown to achieve better adherence [7]. Education outside the classroom (EOtC) is be such an approach [17, 18].

EOtC has received widespread attention in the face of COVID-19 as a means to reduce school-based transmission [19]. Recent evidence suggests that EOtC in general, and perhaps even more so when conducted in nature, holds benefits for health and wellbeing [20–22]. Pupils have been observed to engage in more PA during EOtC compared to usual classroom-based teaching [23–25] as well as report higher school wellbeing [26, 27], school motivation [28], and academic achievement [29]. Therefore EOtC has been characterised [17, 18] as an intersectoral [30] and integrated ‘win-win’ opportunity for both health and education sectors, i.e., an ‘add-in’ approach to school-based PA promotion [7].

In EOtC, teaching sessions are relocated from the classroom to, for example, public open spaces, societal institutions, or outdoor spaces on school property. Examples include the use of the properties in the local neighbourhood to teach geometry (e.g., identifying square or triangular objects), writing poems in and about the city, investigating local history in the neighbourhood, doing STEM education at science centers, or learning about biological and chemical processes through school gardening [31]. The aim of EOtC is to promote learning and wellbeing through practical exercises and the use of one’s body and senses in authentic situations [32, 33]. EOtC often involves teaching activities that require active transportation to and around places outside the school buildings. EOtC is further characterised by the pedagogical and didactical elements of pupil-led approaches, collaborative, action-centred, experiential, inquiry-based and thematic, play-based learning processes, and movement [34, 35]. EOtC involves outdoor teaching explicitly aligned with and contributing to teachers’ obligations [17, 21, 34], often in a cross-curricular approach.

The pedagogical approaches that underpin EOtC are thought to stimulate investigative behaviours based on pupils’ curiosities alone or in groups, and thereby foster experiences with autonomy as well as relatedness. Tangible and practical work allows an experience of being competent in other fields than, e.g., reading and writing. EOtC pedagogy thus aligns with Self-Determination Theory’s (SDT) [36, 37] perspective on motivation and wellbeing [36] in enabling experiences of autonomy and a sense of competency in close relations with teachers and peers [38–40]. SDT is one of the leading wellbeing and motivation theories in the world and widely used in the context of education [41, 42]. SDT represents a broad framework for the study of human motivation, flourishing, and wellbeing. At the centre of the theory is the individual’s basic psychological need for *autonomy, competence*, and *relatedness*, which when fulfilled has been shown to result in wellbeing and the most self-determined forms of motivation [43–45]. SDT’s usefulness for EOtC interventions was confirmed in the so-called TEACHOUT study [28, 38] and has also been used as a central theory to explain the benefits of EOtC in other studies [46, 47].

The TEACHOUT study was conducted in the school year 2014–2015 [38]. The study was a quasi-experimental investigation of the associations of EOtC with PA, wellbeing, and learning among pupils in grades 3-6in Danish public schools. The study was the first large-scale quantitative multi-outcome investigation of EOtC, and a trailblazer study in its field [21, 48]. The TEACHOUT intervention included a two-day training course for teachers followed by them implementing EOtC at least five hours a week, divided in 1–2 weekly sessions, for one school year [49]. The study showed that regular practice of EOtC was positively associated with device-based measured PA, with the boys seeming to accrue more moderate-to-vigorous PA (MVPA) and girls more light PA (LPA) [23, 24]. The TEACHOUT study also showed positive effects on pro-social behaviour [26] and in-class social relations [27], motivation for school [28], and reading competence [29]. However, this previous research on effects of EOtC has methodological limitations that need to be addressed. PA was only measured once during the intervention, making the design of the PA evaluation cross-sectional and unable to exclude influences of extraneous impacts on outcomes. This made it impossible to ascertain whether it was EOtC itself that lead to improvements in PA over and above the usual practices in the schools. In addition, the study sample did not allow for investigation of subsamples of pupils, e.g., pupils with overweight or inactive girls, who might attain different benefits of EOtC. Furthermore, the collected data did not allow for investigation of compensatory movement behaviours outside the school day [50] or potential adverse effects (e.g., subgroups who do not feel comfortable or safe outside). Furthermore, there is a need for investigation of which pedagogical and didactical elements of the EOtC that mediate and moderate potential effects.

Therefore, on the foundation of both the merits and limitations of the original TEACHOUT study, this study protocol describes the MOVEOUT study. The MOVEOUT study will provide a more robust and reliable evaluation of the potentials and mechanisms of EOtC. Evidence from MOVEOUT will allow formulation of guidelines for practice and policy with details about the pedagogical and didactical elements of EOtC, which need to be emphasised in the ongoing expansion of EOtC.

### Aims

**Fig. 1:**
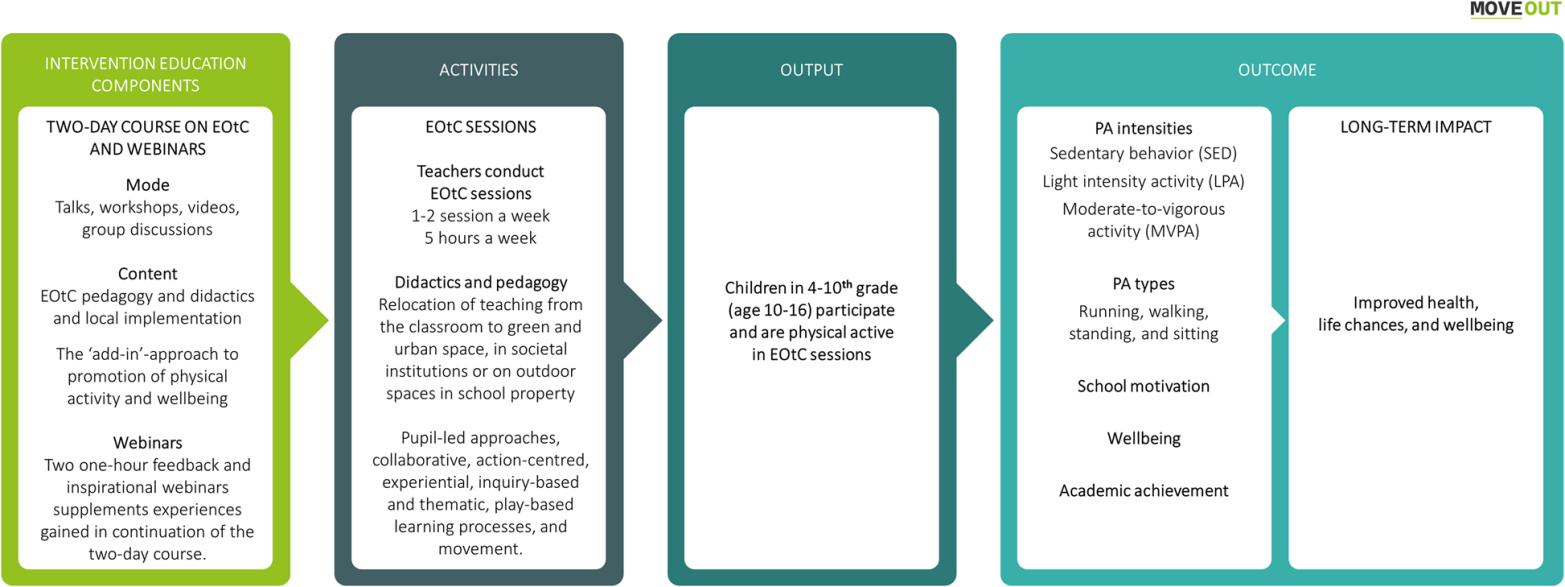
The MOVEOUT study theory of change

This study protocol outlines the design and methods of the MOVEOUT study. The objectives of the MOVEOUT study are to investigate the efficacy and mechanisms of the *TEACHOUT intervention* [49] (hereafter, the intervention) on Danish grade 4–10 pupils’ (aged 10–16 years) school-based and overall PA behaviours, school motivation, wellbeing, and academic achievement over the course of one school year in comparison to the schools’ usual practice. The schools’ usual practice typically involves indoor classroom-based activities. The intervention is the same well-described and manualised EOtC intervention that was investigated in the abovementioned quasi-experimental study, TEACHOUT [49]. See the MOVEOUT study Theory of Change, Fig. 1.

### Sub-studies and hypotheses

**Fig. 2:**
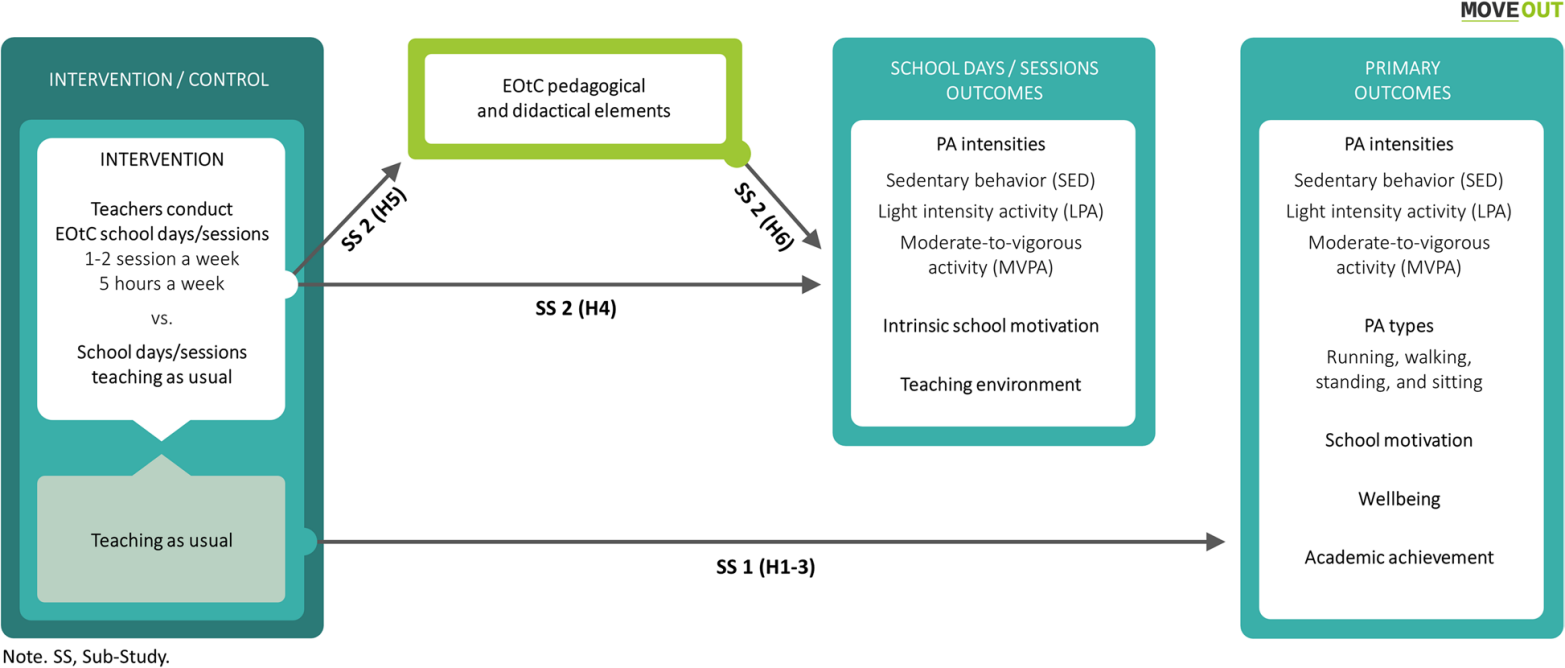
Illustrations of the hypotheses of the two sub-studies

The study objectives will be reached through two sub-studies – an efficacy and a mechanism study – testing six main hypotheses, which are illustrated in Figure 2.

#### Sub-study 1

Effects of EOtC (see full pre-registered protocol for H1 and H2 ([51]).

- H1 Pupils in the intervention group will retain higher PA intensities compared to the control group, specifically a) more MVPA, b) more LPA, and c) less SED time (referred to in the following only as PA intensities).
- H2 Pupils in the intervention group will retain a) more running, b) more walking, c) more standing, and d) less sitting time compared to the control group.
- H3 Pupils in the intervention group will achieve higher school motivation, wellbeing, and academic achievement compared to the control group.

#### Sub-study 2

Mechanisms of EOtC. The unit of analysis is schooldays or sessions.

- H4 EOtC schooldays or sessions will be associated with higher PA intensities, higher levels of intrinsic motivation, and more positive perceptions of the teaching environment compared to usual schooldays or sessions.
- H5 The pedagogical and didactical elements during EOtC sessions will be different from the pedagogical and didactical elements during usual sessions.
- H6 The expected association between EOtC and PA intensities, intrinsic motivation, and perceptions of the teaching environment will be mediated by the pedagogical and didactical elements of the EOtC sessions.

## Methods

The following paragraphs outlines the methods of the MOVEOUT study in accordance with the SPIRIT (Standard Protocol Items: Recommendations for Interventional Trials) guidelines [52]. Recruitment, data collection, and intervention training was commenced prior to publication of the protocol (but after registration to ClinicalTrials), and parts of the sections pertaining to past events are therefore written in past tense below.

**Fig. 3:**
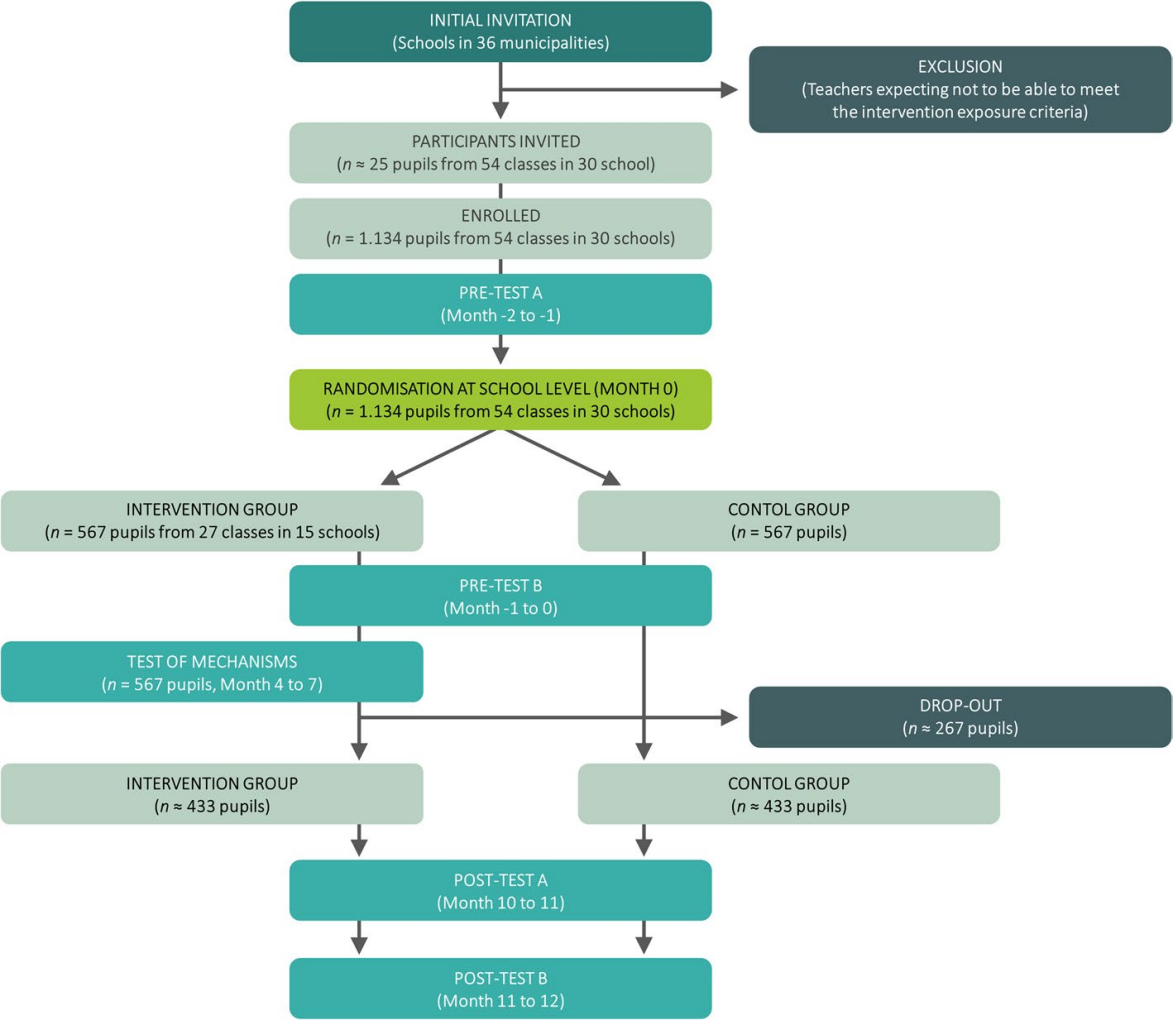
Participation allocation flowchart of the MOVEOUT study according to SPIRIT

The MOVEOUT study is a cluster-randomised trial with randomisation occurring at school level. Elementary schools with one or more classes enrolled was randomly assigned to the intervention or control groups. The intervention consisted of a two-day training course on EOtC given to schoolteachers followed by the teachers applying EOtC for at least five hours a week with their class, in 1–2 weekly sessions for one school year. The training course was supplemented by two one-hour inspirational webinars during the first half of the school year (see TIDieR checklist for more detailed description [49]). The schools assigned to the control group will continue their teaching as usual for one school year upon which they will also receive the intervention. See timeline of enrolment, allocation, intervention, and data collection according to SPIRIT in Table 1 and Fig. 3.

**Table 1:** Participant timeline according to SPIRIT

	STUDY PERIOD							
					Implementation			
	Enrolment	Pre-test A	Pre-test B	Allocation		Post-test A	Post-test B	Close-out
Time point	-18 mo.	-2 mo.	-1 mo.	0 mo.	4 mo.	10 mo.	11 mo.	14 mo.
**ENROLMENT**	x							
Eligibility screen	x							
Informed consent	x							
Allocation (cluster)				x				
**INTERVENTIONS**								
Two-day training course				x				
Two one-hour inspirational webinars					x			
Applying EOtC > five hours pr. week in 1–2 weekly sessions					x	x	x	x
**MEASUREMENTS**								
* Participants*								
Pupil socioeconomic status (parent survey)	x							
Pupil health status (parent survey)	x							
Implementer descriptives (teacher survey)	x							
* Pre- and post-measures*								
PA behaviours (pupil device based, seven days)		x	x			x	x	
School motivation (pupil questionnaire)			x				x	
Wellbeing (pupil questionnaire)			x				x	
Academic achievement (pupil tests)			x				x	
* School days/sessions measures (two EOtC and two non-EOtC schooldays, IG only)*								
PA behaviours (pupil device based, session)					x			
School motivation (pupil survey, school day)					x			
Learning environment (pupil survey, school day)					x			
Didactical and pedagogical elements (observation, school day)					x			
* Implementation*								
Degree and characteristics of the EOtC (teacher questionnaire, weekly)					x	x	x	x
Adaption, acceptability, and local supportive structures (teacher interview, IG only)						x		
* Background descriptive measures*								
Socioeconomic status (survey, parent/legal guardian)		x						
Child health status (survey, parent/legal guardian)		x						
Teacher’s previous work and EOtC experience (survey)			x					

*EOtC *Education outside the classroom, *IG *Intervention group. *Mo *Month(s), *PA *Physical activity

### Setting and context

The study is conducted in Denmark in typical Danish elementary schools (for 6- to 16-year-old children). Regular practice of EOtC has increased markedly in Denmark within the last twenty years with at least 19.5% of all schools reporting EOtC in some of their classes in 2019 [53].

During the COVID-pandemic, Denmark was among the first European countries to reopen schools by allowing teaching in primary school classes to take place outdoors [54]. This fostered a further boom in schools and teachers seeking supportive guidelines for outdoor classroom management and ideas of EOtC [55]. In response, various educational actors and organisations developed and offered the needed knowledge, extending the general supply of freely available materials, e.g., Children & Nature – Denmark [56].

### Recruitment and participants

We aimed to recruit ~ 54 classes (~ 81 teachers) from 30 Danish general (non-special need) education elementary schools; on average 1.8 classes per school, 1–2 teachers and 21 pupils per class, with a total of 1.134 pupils aged 10–16 years (see Fig. 3). Pupils with noteworthy health problems, reported by their parents or legal guardian, teachers, or investigators will be excluded from the analyses.

Schools in municipalities with larger proportions of citizens with lower levels of education than the average for the region were strategically invited for enrolment in the study. Schools located in the Capital Region and Region Zealand were invited to begin with. Thereafter, schools in Region of Southern Denmark were invited, and finally invitations were distributed nationally via teacher networks. School classes were not allowed to be involved in other school development or research projects.

Recruitment and enrolment started around two years before the implementation of the intervention and continued until randomisation (month − 18 to 0, see Table 1). Teachers were recruited via both municipal school directors and direct contact to local school principals. Municipalities and schools were initially contacted via e-mail and non-responders were then contacted via telephone. Online information meetings were held with school principals and teachers. Participating teachers informed the parents and were provided with written material available in Danish and English, and supplementary video material introducing the study (see Supplementary Information, Additional files 1, 3, and 4). Parents were given a letter with additional information about the study’s rationales and background, and an analogue consent form. The recruitment process was supported by general information on the MOVEOUT study project website (www.moveoutstudy.dk) [57].

### Intervention

**Fig. 4:**
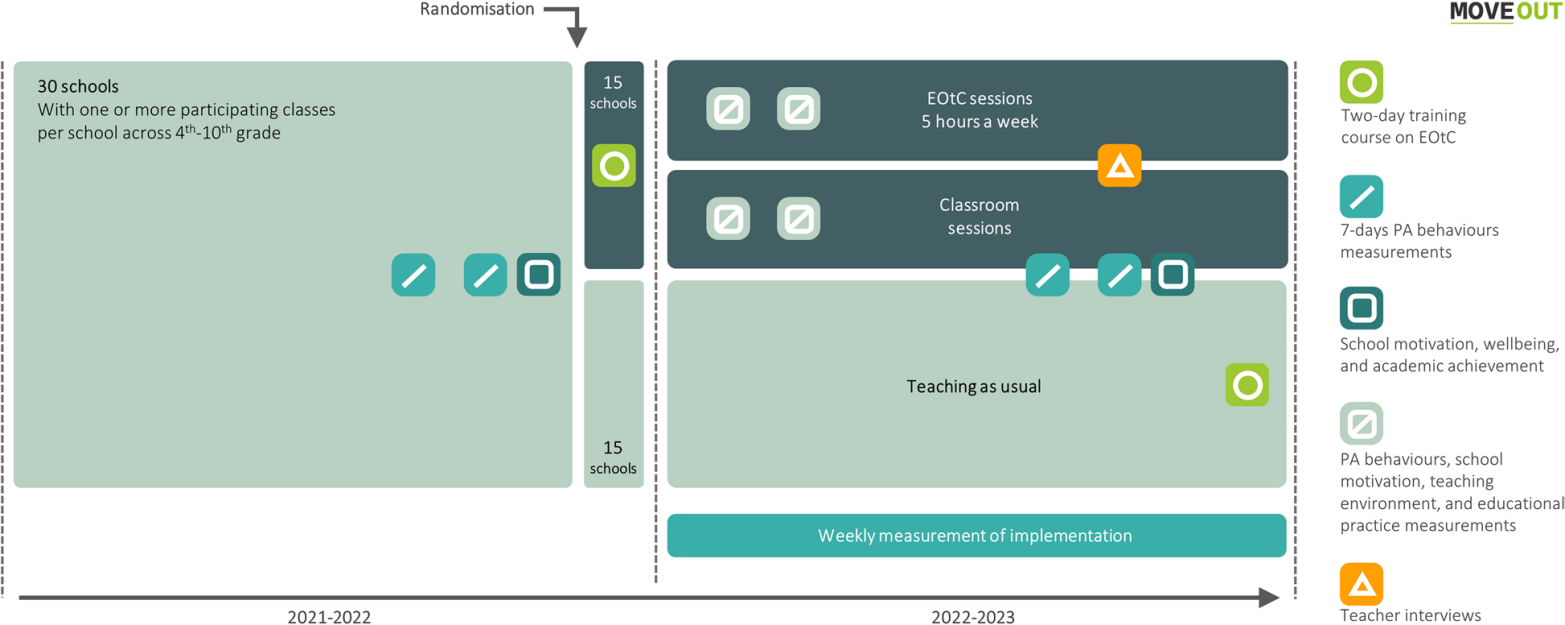
The MOVEOUT study design

The intervention is based on the TEACHOUT intervention [49]. It is comprised of a two-day training course (13 h in total) on EOtC for teachers who afterwards implement EOtC in 1–2 weekly sessions, a total of at least five hours per week, for one schoolyear (in this study, August 2022 to June 2023, Month 4 to 14, see Fig. 4). The EOtC sessions in the schools are delivered by one or more of the teachers across different school subjects in various places and settings outside the school buildings.

The course was structured to provide the teachers with knowledge and understanding of the theory and practice of EOtC. The course was taught by expert EOtC teachers and teacher education specialists and included theoretical lectures, supplemented by illustrative examples in workshops and videos, and plenum discussions on local implementation. The training course took place on the 28th and 29th of April 2022 (after randomisation, Month 0, see Table 1). The course was supplemented by two one-hour inspirational webinars during the first half of the school year (in this study, in September and November 2022, i.e., during Month 4 to 9, see Table 1).

Teachers in the control group received the two-day training course on EOtC in April 2023, close to the end of the intervention group implementation closeout; however, early enough for them to use the course content in their planning of the following school year. Also, they will receive the two one-hour inspirational webinars in the autumn 2023.

### Randomisation

Randomisation was performed by PE and MB on 1st of April 2022 (Month 0, see Fig. 4), and was made public on YouTube (see Supplementary Information, Additional file 1). Immediately after, the enrolled schools and teachers were informed by e-mail about the result of the randomisation process.

To ensure a balanced representation of grade levels across the intervention and control groups, stratified block randomisation in a 1:1 ratio was used (i.e., schools with participating classes in grade 4–6 and schools with classes 7–10 were randomised block wise in RStudio using the ‘dplyr’ and ‘readxl’ packages).

Due to the type of the intervention, it was not possible to blind the participants to group allocation. However, allocation levels will be coded before analyses and the analysts will not be informed about the codes before the final analyses have been performed.

### Sub-study 1: effects of EOtC

#### Design

Effects of EOtC will be measured in a between-subjects design. PA measurements pre- and post-intervention will be collected in two intervals each (Test A and B) to account for seasonal variations. Data collection of the first PA pre-intervention measure commenced before randomisation and continued until just before randomisation (Pre-test A, Month -2 to -1, see Table 1). The second PA pre-measure was collected after randomisation and before the implementation of the intervention (Pre-test B, Month -1 to 0). School motivation, wellbeing, and academic achievement were measured at pre-test B.

The first PA post-measure data collection will commence during the end of the school year (Post-test A, Month 10 to 11, see Table 1). The second PA post-measure data collection will be assessed immediately before finalisation of the intervention (Post-test B, Month 11 to 12, see Table 1). School motivation, wellbeing, and academic achievement will be measured at post-test B.

#### Outcome measures

*School-based and overall PA behaviours* will be measured with Axivity® AX3 accelerometers (see full pre-registered study description of the investigation of PA behaviours [51]). All acceleration data will be processed in Matlab (Version 9.9.0 R2020b, Mathworks Inc., Natick, Massachusetts, US), which includes resampling, generating ActiGraph counts [58], identification of non-wear, and summarising the subjects’ time spent in different PA intensity domains and with types of PA behaviour as running, walking, standing, and sitting. Non-wear periods is identified from both acceleration and temperature using the method described in the study by Rasmussen et al. [59]. Pupils should have valid data from at least one pre-intervention PA measurement.

*School motivation* is measured using the Academic Self-Regulation Questionnaire (SRQ-A) [60]. The SRQ-A is a domain-specific 32-item self-report questionnaire developed for measuring the level of autonomy relative to doing different types of schoolwork among pupils in late primary and lower-secondary school. In this study, the items from the homework domain are omitted as not every school in Denmark uses homework.

*Wellbeing* will be measured using two different scales, the overall score from the KIDSCREEN-27 questionnaire and the pro-social behaviour scale of the Strengths and Difficulties Questionnaire (SDQ). KIDSCREEN-27 is the 27-item version of the KIDSCREEN Health-Related Quality of Life (HRQoL) questionnaire which measures the subjective health, wellbeing, and health-related quality of life of children aged 8–18 years [61]. The pro-social behaviour scale of the SDQ measures resources and functioning in terms of social skills and competences of children aged 11–17 years [62, 63], although high reliability was reported for use in pupils aged 10 years in a similar EOtC intervention study [26].

*Academic achievement* will be measured with two scales measuring reading competence and math skills. Reading competence will be measured with the validated age-adapted text reading test (Tekstlæseprøve, Grade 3–7; Tekstforståelse Udsagn A [Text comprehension Statement A], Grade 8–10) by Hogrefe [64]. Math skills will be measured with the validated age-adapted mathematical basic skills test (MG, Matematik Grunglæggende [Mathematics Basics]) by Hogrefe [65].

#### Sample size

Three classes were expected to drop-out during the intervention as per observations in the TEACHOUT study [38]. We further expected a sample size reduction by approximately 20% of the pupils due to drop-out and invalid accelerometer data (equivalent to four pupils per class). We expected an analytical sample of ~ 30 schools, ~ 51 classes, and ~ 867 pupils in total. The expected analytical sample allows 95% power to identify a small to medium effect size using a one tailed test (Cohen’s d = 0.374 95% CI [0.187,0.561]). Calculations accounted for intraclass correlations (ICC) derived from the TEACHOUT dataset of 0.046 within classes and 0.035 within schools. Power calculations were conducted using R package PowerUpR [66].

#### Statistical analyses

Estimation of intervention effects (H1, H2, and H3 of Sub-study 1) will be computed using a ‘per-protocol’ analysis [67]. All classes in the intervention group not adhering to the classification of ‘regular EOtC’ (an average of > 150 min of weekly EOtC over the school year) and all classes in control group practicing too high amount of EOtC (average of > 150 min of weekly EOtC over the school year) will be excluded. These analyses will be complemented with sensitivity analyses, following the principle of ‘intention-to-treat’ [67] which will include all schools in the analyses irrespective of their adherence to the intervention or control condition.

Linear mixed models (LMM) will be used to analyse how post-test scores varies as a function of whether the pupils were assigned to the intervention or the control group while taking into account pre-test scores, sex, age, and random effects of classes and schools. Please, see the H1 and H2 pre-registration for further information on the pre-processing steps and statistical modelling used for these data [51]. The base model used to assess H3 (effects on school motivation, wellbeing, and academic achievement) will include baseline as a covariate and school and class ID as random factor, and group allocation as the fixed effect of primary interest. Follow-up stepwise modelling will be used to investigate the contribution of previously shown confounders, i.e. primarily the number of weekly EOtC sessions [26], age, and sex [23, 68, 69], to the base models. All sub-studies will be reported following the CONSORT statements and flowchart [70].

### Sub-study 2: mechanisms of EOtC

#### Design

We will investigate the mechanisms of EOtC in a within-subjects design. Pupils in the intervention group will have their PA intensities measured during two randomly chosen EOtC and two usual school days in the first half of the implementation period (Month 4 to 7, see Table 1). The pupils will answer a school motivation and learning environment questionnaire at the end of each day. The pedagogical and didactical elements will be observed during sessions.

#### Outcome measures

*PA intensities* will be measured with wrist mounted Axivity® AX3 accelerometers during school sessions and data will be processed as in Sub-study 1.

*Motivation for the teaching activities* will be measured at pupil-level with the Interest/Enjoyment (3-items), Perceived Choice/autonomy (3-items), and Perceived Competence (3-items) sub-scales of the Intrinsic Motivation Inventory (IMI) [71]. The IMI is an activity-specific measure for intrinsic motivation and basic needs satisfaction [36, 72] specially designed to be distributed immediately after an activity. Experiences of Relatedness was measured with three items inspired by the relatedness satisfaction subscales of the Basic Psychological Need Satisfaction Scales, Basic Psychological Need Satisfaction and Frustration Scale (BPNSNF) [73] and Psychological Need States in Sport-Scale (PNSS-S) [74].

*Learning environment perception* will be measured at pupil level with the Learning Rating Scale (LRS) [75]. LRS is a 4-item scale answered on a 10 cm visual analogue scale and assesses the pupils’ perceptions of how much they learn at school, how well they are getting along, and how well the teaching methods fit them.

*Pedagogical and didactical elements* will be quantified with an adapted version of the elementary school teaching practice observation scheme, Einblicknahme in die Lehr- und Lernsituation [76], using items 1–35 of the Danish version [77]. The observation scheme includes five pedagogical and didactical themes including class management; recognition and motivation; structure and consolidation; autonomy and participation; and differentiation. The scheme will be supplemented by six EOtC-specific [32, 35] and two lesson movement integration items [78, 79], as well as measures of what type of place the teaching takes place outside the school buildings [39]. These eight items will be used as a sixth theme to measure action-centred, experiential, inquiry-based, play-based learning processes theorised to pertain in particular to EOtC.

#### Statistical analyses

PA intensities during EOtC and usual school sessions will be analysed in three LMM’s with sessions as unit of analysis to investigate each PA intensity (i.e., MVPA, LPA, and sedentary time) as a function of whether the pupils have taken part of a EOtC or usual school session (H4). The base model will include school days, nested in observations nested in pupils, nested in school class as random factors, and EOtC/usual sessions as the fixed effect.

Differences in pedagogical and didactical practice will be analysed in six LMM’s (H5, one for each of the elements) with sessions as unit of analyses to investigate pedagogical and didactical practice as a function of the degree to which the practice occurred during a EOtC or usual session. The base model will include school days, nested in school class as random factor, and EOtC/usual sessions as the fixed effect. With school days as unit of analysis, two LMM’s (H5) will be used to analyse how school motivation and learning environment perceptions vary as a function of whether the pupils have taken part of a EOtC or usual school days. The base model will include observations in pupils, nested in school classes as random factors, and EOtC/usual school day as the fixed effect.

Follow-up stepwise modelling will be used to investigate if previously shown confounders, i.e., length of EOtC sessions [26], age, and gender [23, 68, 69], will improve the PA intensity, school motivation, and learning environment perception base models.

The mechanisms of EOtC (H6) will be analysed in individual secondary structural equation models (SEM) for each of the three proximal outcomes, i.e., session PA, school motivation, and learning environment perception. In all three models, EOtC/usual schooldays or session will be included and the types of EOtC pedagogy applied in the session or schooldays will be investigated as mediators [80].

### Evaluation of implementation

#### Design

Adherence to the intervention will be investigated in a repeated design with weekly monitoring of EOtC practised during the intervention in the intervention group and control group (Month 4 to Month 14, see Table 1). Quality of delivery, variations, facilitating and limiting contextual factors at school, local, and municipality level, and teachers’ acceptability of the intervention will be investigated through semi structured interviews [81] with intervention group teachers taking place in the second half of the implementation period (Month 10, see Table 1).

#### Outcome measures

Monitoring of the intervention, namely the degree and the characteristics of the EOtC implementation, will be measured with a short self-report teacher questionnaire distributed weekly to teachers by SMS every Friday during the implementation period. In the previous TEACHOUT study, non-intended EOtC implementation in the control group was found to be 1.64 h a week [39]. The questionnaire assesses the amount, place, mode of transportation, and school subjects used during EOtC sessions. The questionnaire is based upon the existing and validated EOtC implementation monitoring tool from the TEACHOUT study [38, 39].

### Background descriptive measures

Perceived child health status and data on socioeconomic status (parents’ or legal guardian’s education level and occupational status) will be collected using the Danish Occupational Social Class Measurement [82] with the pupils’ parents (or legal guardian) (Month -2, see Table 1).

Quantitative data about teachers previous work experience and previous experience with their use of EOtC will be collected from the intervention and control group teachers (Month 0, see Table 1).

### Data processing

All analyses will be processed in either RStudio or IBM® SPSS® Statistics with the level of statistical significance for all tests set to < 0.05.

### Trial status

Recruitment and enrolment were initiated two years before the implementation of the intervention and continued until randomisation (Month -18 to 0, see Table 1). The trial is progressing according to the time plan.

## Discussion

EOtC holds promise for improved health and education if implemented regularly and extensively. The threshold, if there is one, for ‘how much’ EOtC is required for health and education gains is uncertain, but previous experience and findings point to five hours a week as a minimum. However, this current understanding of EOtC rests on findings from previous studies (e.g. [24, 27, 29]) with inherent risks of bias. The MOVEOUT study investigates the effects of the intervention developed and conducted in the TEACHOUT study [38] with updated and strengthened design and measures. The foremost strengths and limitations of the MOVEOUT study are discussed below.

The MOVEOUT study aims to reach more solid conclusions of the effects and mechanisms of EOtC using randomised group allocation, quantification of the pedagogical and didactical elements that are posited to carry effects, and extensive monitoring. The study will not only be able to confirm or disconfirm previous assertions relating to EOtC, but also contribute to a deeper understanding of the so-called active ingredients. Pending the findings, the combination of these data sources provides considerable prescriptive potential for practice and policy.

The use of randomisation will minimise any influences on effect measures of teachers’ having different initial interest in and abilities for EOtC. It can be expected that being randomised to do what you as a teacher have hoped for when signing up for the study, i.e., being assigned to the intervention group, may affect pupil outcomes positively. It may also be that the teachers ending in the control group may not refrain from using EOtC. Therefore, the collected weekly monitoring data about EOtC practise among all classes in the project is important data to include in the analysis.

The MOVEOUT study enables an effect evaluation of EOtC-induced PA behaviours by having pre-intervention measures of PA in order to adjust post-intervention PA for baseline PA. We further use two pre-intervention and measures and two post-intervention measures to account for impacts of seasonal changes in weather. Such a design is seldom applied in the studies investigating the influence of comparable interventions on PA [21, 83], and will provide a more valid analysis of the intervention effect that the previous study on the effects of EOtC on PA [23].

Effectiveness and efficacy studies of educational interventions often fail to report whether and how the intervention was monitored [84, 85]. Such a lack of monitoring is also evident in outdoor activity programs [86] and is a general limitation for outdoor learning programs [87]. Lack of intervention monitoring, leads to a ‘black box’ approach to intervention studies, where implementation is not evaluated [88]. In the MOVEOUT study, such an approach is avoided by combining the quantitative monitoring with on-site observation on school days involving EOtC sessions and usual schooldays. Combined with school day specific assessment of PA and school motivation and learning environment perceptions, monitoring and observations enable an investigation of key mechanisms of EOtC.

### Supplementary Information

**Additional file 1: Table SA1.** The MOVEOUT study video materials.

**Additional file 2.** MOVEOUT SPIRIT 2013 Checklist: Section, item number, and manuscript page number.

**Additional file 3. **Information about the project and the processing of data (parents).

**Additional file 4. **Information about the project and the processing of data (teachers).

## Data Availability

Anonymised participant-level data can be made available upon reasonable request to the corresponding author, such as verification of the research results. To comply with the European General Data Protection Regulation, and to accommodate article 10 of the Danish Act on Data Protection anonymised participant-level data will be made available on the Open Science Framework five years after the main publication of this study (link available in the reference list [51]).

## Abbreviations

BMI: Body Mass Index
EOtC: Education outside the classroom
EUR: Euro currency
HRQoL: Health-Related Quality of Life
LPA: Light-intensity physical activity
MI: Movement integration
MVPA: Moderate-to-vigorous physical activity
PA: Physical activity
RCT: Randomised Controlled Trial
SDQ: Strengths and Difficulty Questionnaire
SDT: Self-Determination Theory
SPIRIT: Standard Protocol Items: Recommendations for Interventional Trials
SRQ-A: Academic Self-Regulation Questionnaire

## References

[1] Basch CE (2011). Healthier students are better Learners: a missing link in School Reforms to close the achievement gap. J Sch Health.

[2] Case A, Fertig A, Paxson C (2005). The lasting impact of childhood health and circumstance. J Health Econ.

[3] Suhrcke M, de Paz Nieves C. The impact of health and health behaviours on educational outcomes in high-income countries: a review of the evidence. World Health Organization. Regional Office for Europe; 2011 [cited 2023 Apr 5]. vi, 35 p. Available from: https://apps.who.int/iris/handle/10665/345467.

[4] Cutler D, Lleras-Muney A (2014). Health and Education. Encyclopedia of Health Economics.

[5] Inchley J, Currie D, Young T, Samdal O, Torsheim T, Augustson L et al. Growing up unequal: gender and socioeconomic differences in young people’s health and well-being. Health Behaviour in School-aged children (HBSC) study: international report from the 2013/2014 survey. Health Policy Child Adolesc. 2016;(7). Available from: https://apps.who.int/iris/rest/bitstreams/1241244/retrieve.

[6] Strum R (2005). Childhood obesity—what we can learn from existing data on societal trends, part 1. Prev Chronic Dis.

[7] Bentsen P, Bonde AH, Schneller MB, Danielsen D, Bruselius-Jensen M, Aagaard-Hansen J. Danish ‘add-in’ school-based health promotion: integrating health in curriculum time. Health Promot Int. 2018 [cited 2018 Nov 30]; Available from: https://academic.oup.com/heapro/article-abstract/35/1/e70/5218996. 10.1093/heapro/day095 30500915

[8] OECD. The future of education and skills: Education 2030. 2018 [cited 2019 Jun 12]. Available from: https://www.oecd.org/education/2030/E2030%20Position%20Paper%20(05.04.2018).pdf.

[9] Lee IM, Shiroma EJ, Lobelo F, Puska P, Blair SN, Katzmarzyk PT (2012). Effect of physical inactivity on major non-communicable diseases worldwide: an analysis of burden of disease and life expectancy. The Lancet.

[10] Biddle SJH, Ciaccioni S, Thomas G, Vergeer I (2019). Physical activity and mental health in children and adolescents: an updated review of reviews and an analysis of causality. Psychol Sport Exerc.

[11] Barbosa A, Whiting S, Simmonds P, Scotini Moreno R, Mendes R, Breda J (2020). Physical activity and academic achievement: an Umbrella Review. Int J Environ Res Public Health.

[12] Toftager M, Brønd JC. Fysisk aktivitet og stillesiddende adfærd blandt 11-15-årige: National monitorering med objektive målinger [Physical activity and sedentary behavior among 11-15-year-olds: National monitoring with objective measurements]. National Board of Health; 2019. Available from: https://www.sst.dk/-/media/Udgivelser/2019/Fysisk-aktivitet-og-stillesiddende-adfaerd-blandt-11-15-aarige-monitorering-m-objektive-maalinger.

[13] Patalay P, Gage SH (2019). Changes in millennial adolescent mental health and health-related behaviours over 10 years: a population cohort comparison study. Int J Epidemiol.

[14] Norris E, Shelton N, Dunsmuir S, Duke-Williams O, Stamatakis E (2015). Physically active lessons as physical activity and educational interventions: a systematic review of methods and results. Prev Med.

[15] Routen AC, Chalkley AE, Sherar LB (2017). Getting a GRIP (getting research into practice) on movement integration in the school classroom. Phys Ther Rev.

[16] Erwin H, Fedewa A, Beighle A, Ahn S (2012). A quantitative review of physical activity, health, and learning outcomes associated with classroom-based physical activity interventions. J Appl Sch Psychol.

[17] Bentsen P, Bølling M, Mygind L, Schneller MB, Stevenson MP, Mygind E (2019). Greening education: outdoor learning in natural settings as an ‘add-in’ holistic school-based health promotion approach for children and young people. Physical activity in natural settings: green exercise and blue mind.

[18] Bentsen P, Mygind L, Elsborg P, Nielsen G, Mygind E. Education outside the classroom as upstream school health promotion: ‘adding-in’ physical activity into children’s everyday life and settings. Scand J Public Health. 2022;50(3):303–11. 10.1177/1403494821993715 33624553

[19] Quay J, Gray T, Thomas G, Allen-Craig S, Asfeldt M, Andkjaer S (2020). What future/s for outdoor and environmental education in a world that has contended with COVID-19?. J Outdoor Environ Educ.

[20] Mann J, Gray T, Truong S, Brymer E, Passy R, Ho S et al. Getting Out of the Classroom and Into Nature: A Systematic Review of Nature-Specific Outdoor Learning on School Children’s Learning and Development. Front Public Health. 2022 [cited 2023 Mar 17];10. Available from: 10.3389/fpubh.2022.877058. 10.3389/fpubh.2022.877058 PMC9149177 35651851

[21] Becker C, Lauterbach G, Spengler S, Dettweiler U, Mess F (2017). Effects of regular classes in Outdoor Education Settings: a systematic review on students’ learning, Social and Health Dimensions. Int J Environ Res Public Health.

[22] Browning MHEM, Rigolon A (2019). School Green Space and its impact on academic performance: a systematic literature review. Int J Environ Res Public Health.

[23] Schneller MB, Duncan S, Schipperijn J, Nielsen G, Mygind E, Bentsen P (2017). Are children participating in a quasi-experimental education outside the classroom intervention more physically active?. BMC Public Health.

[24] Schneller MB, Schipperijn J, Nielsen G, Bentsen P (2017). Children’s physical activity during a segmented school week: results from a quasi-experimental education outside the classroom intervention. Int J Behav Nutr Phys Act.

[25] Bølling M, Mygind E, Mygind L, Bentsen P, Elsborg P (2021). The association between education outside the classroom and physical activity: differences attributable to the type of space?. Children.

[26] Bølling M, Niclasen J, Bentsen P, Nielsen G (2019). Association of Education outside the Classroom and Pupils’ Psychosocial Well-being: results from a School Year implementation. J Sch Health.

[27] Bølling M, Mygind E, Pfister G, Nielsen G (2019). Education outside the classroom and pupils’ social relations? A one-year quasi-experiment. Int J Educ Res.

[28] Bølling M, Otte CR, Elsborg P, Nielsen G, Bentsen P (2018). The association between education outside the classroom and students’ school motivation: results from a one-school-year quasi-experiment. Int J Educ Res.

[29] Otte CR, Bølling M, Stevenson MP, Nielsen G, Bentsen P, Ejbye-Ernst N (2019). Education outside the classroom increases children’s reading competencies: results from a one-year danish quasi-experimental study. Int J Educ Res..

[30] World Health Organization. Intersectoral governance for health in all policies: structures, actions and experiences. World Health Organization. Regional Office for Europe; 2012. Available from: https://apps.who.int/iris/bitstream/handle/10665/326430/9789289002813-eng.pdf.

[31] Bølling M, Barfod KS, Elsborg P, Mygind L, Mygind E, Bentsen P et al. Udeskole der bevæger – Viden, inspiration og eksempler på undervisningsforløb [Udeskole that moves - Knowledge, inspiration and examples of teaching courses]. Steno Diabetes Center Copenhagen, VIA University College, Center for Klinisk Forskning og Forebyggelse, Center for Børn og Natur & Institut for Geovidenskab og Naturforvaltning, Københavns Universitet.; 2023. Available from: https://centerforboernognatur.dk/projekter/udb/.

[32] Bentsen P, Mygind E, Randrup TB (2009). Towards an understanding of udeskole: education outside the classroom in a danish context. Educ 3–13.

[33] Waite S, Bølling M, Bentsen P (2015). Comparing apples and pears?: a conceptual framework for understanding forms of outdoor learning through comparison of English Forest Schools and danish udeskole. Environ Educ Res.

[34] Bærenholdt J, Hald M, Carter C. Udeskole in Theory and Practice: a danish Approach to Learning outside the Classroom. Dafolo; 2022.

[35] Barfod KS, Daugbjerg P (2018). Potentials in Udeskole: Inquiry-Based teaching outside the Classroom. Front Educ.

[36] Deci EL, Ryan RM (2000). The what and why of goal pursuits: human needs and the self-determination of behavior. Psychol Inq.

[37] Deci EL, Ryan RM. Intrinsic motivation and self-determination in human behavior. 1985 [cited 2016 Jan 26]. 10.1007/978-1-4899-2271-7.

[38] Nielsen G, Mygind E, Bølling M, Otte CR, Schneller MB, Schipperijn J (2016). A quasi-experimental cross-disciplinary evaluation of the impacts of education outside the classroom on pupils’ physical activity, well-being and learning: the TEACHOUT study protocol. BMC Public Health.

[39] Bølling M. Udeskole og børns trivsel. Et kvasi-eksperimentelt interventionsstudie af sammenhængen mellem ét års regelmæssig eksponering for udeskole og børns psykologiske trivsel, skolemotivation og sociale relationer [Education outside the classroom and children’s well-being. A quasi-experimental intervention study of the association between one year of regular exposure to education outside the classroom and children’s psychological well-being, school motivation, and social relations.] [PhD Thesis]. [Copenhagen]: University of Copenhagen, Department of Nutrition, Exercise and Sports; 2018. Available from: https://nexs.ku.dk/english/research-files/phd/Mads-Bolling_Udeskole-phd-web.pdf.

[40] Dettweiler U, Lauterbach G, Mall C, Kermish-Allen R. Fostering 21st Century Skills Through Autonomy Supportive Science Education Outside the Classroom. In: Jucker R, von Au J, editors. High-Quality Outdoor Learning: Evidence-based Education Outside the Classroom for Children, Teachers and Society. Cham: Springer International Publishing; 2022 [cited 2023 Mar 17]. p. 231–53. 10.1007/978-3-031-04108-2_13.

[41] Taylor G, Jungert T, Mageau GA, Schattke K, Dedic H, Rosenfield S (2014). A self-determination theory approach to predicting school achievement over time: the unique role of intrinsic motivation. Contemp Educ Psychol.

[42] Ryan RM, Deci EL. Intrinsic and extrinsic motivation from a self-determination theory perspective: definitions, theory, practices, and future directions. Contemp Educ Psychol. 2020;61;101860.

[43] Owen B, Smith K, Lubans J, Ng DR, Lonsdale JYY (2014). Self-determined motivation and physical activity in children and adolescents: a systematic review and meta-analysis. Prev Med.

[44] Ng JYY, Ntoumanis N, Thøgersen-Ntoumani C, Deci EL, Ryan RM, Duda JL (2012). Self-determination theory Applied to Health Contexts: a Meta-analysis. Perspect Psychol Sci.

[45] Gillison FB, Rouse P, Standage M, Sebire SJ, Ryan RM (2019). A meta-analysis of techniques to promote motivation for health behaviour change from a self-determination theory perspective. Health Psychol Rev.

[46] Dettweiler U, Lauterbach G, Becker C, Simon PA. Bayesian Mixed-Methods Analysis of Basic Psychological Needs Satisfaction through Outdoor Learning and its Influence on Motivational Behavior in Science Class. Front Psychol. 2017;8:2235. 10.3389/fpsyg.2017.02235 PMC5742242 29312080

[47] Mackenzie SH, Son JS, Eitel K (2018). Using outdoor adventure to enhance intrinsic motivation and engagement in science and physical activity: an exploratory study. J Outdoor Recreat Tour.

[48] Jucker R, von Au J. Outdoor Learning—Why It Should Be High up on the Agenda of Every Educator. In: Jucker R, von Au J, editors. High-Quality Outdoor Learning: Evidence-based Education Outside the Classroom for Children, Teachers and Society. Cham: Springer International Publishing; 2022 [cited 2023 Mar 17]. p. 1–26. 10.1007/978-3-031-04108-2_1.

[49] The TEACHOUT intervention TIDieR checklist. Department of Nutrition, Exercise and Sports, University of Copenhagen. ; 2023 [cited 2020 Aug 24]. Available from: https://nexs.ku.dk/english/research/sport-individual-society/embodiment-learning-and-social-change/teachout-english/TEACHOUT_TIDieR.pdf.

[50] Gomersall SR, Rowlands AV, English C, Maher C, Olds TS (2013). The ActivityStat hypothesis: the concept, the evidence and the methodologies. Sports Med Auckl NZ.

[51] Mygind L, Elsborg P, Bølling M, Klinker CD, Melby PS, Andreasen AH et al. Registered Report: Efficacy of education outside the classroom to increase adolescent physical activity. 2022 Sep 21 [cited 2023 Jan 31]; Available from: https://osf.io/jcrvh/?view_only=. 10.1038/s41598-024-79138-z PMC11568335 39548298

[52] Moher D, Chan AW. SPIRIT (Standard Protocol Items: Recommendations for Interventional Trials). In: Guidelines for Reporting Health Research: A User’s Manual. John Wiley & Sons, Ltd. 2014:56–67. 10.1002/9781118715598.ch7.

[53] Barfod K, Ejbye-Ernst N, Mygind L, Bentsen P (2016). Increased provision of udeskole in danish schools: an updated national population survey. Urban for Urban Green.

[54] Melnick H, Darling-Hammond L. Reopening Schools in the Context of COVID-19: Health and Safety Guidelines from Other Countries. Policy Brief. Learning Policy Institute. Learning Policy Institute; 2020 [cited 2020 Aug 25]. Available from: https://eric.ed.gov/?id=ED606555.

[55] Barfod KS (2022). A good thing about this is probably that there’s been more freedom to try some things out’ - danish teachers’ experience of teaching outdoors during the COVID-19 pandemic. J Adventure Educ Outdoor Learn.

[56] Center for Nature and Children Denmark. Hjælp - jeg skal ud med børnene [Help - I have to go out with the children]. 2020 [cited 2023 Mar 17]. Available from: https://centerforboernognatur.dk/projekter/hjaelp/.

[57] Department of Nutrition, Exercise and Sports, University of Copenhagen. 2023. MOVEOUT. A cluster randomized controlled trial of the efficacy and mechanisms of an education outside the classroom intervention on adolescents’ physical activity. Available from: http://www.moveoutstudy.dk/.

[58] Brønd JC, Andersen LB, Arvidsson D (2017). Generating ActiGraph counts from raw acceleration recorded by an alternative monitor. Med Sci Sports Exerc.

[59] Rasmussen MGB, Pedersen J, Olesen LG, Brage S, Klakk H, Kristensen PL (2020). Short-term efficacy of reducing screen media use on physical activity, sleep, and physiological stress in families with children aged 4–14: study protocol for the SCREENS randomized controlled trial. BMC Public Health.

[60] Ryan RM, Connell JP (1989). Perceived locus of causality and internalization: examining reasons for acting in two domains. J Pers Soc Psychol.

[61] Ravens-Sieberer U, Gosch A, Rajmil L, Erhart M, Bruil J, Duer W (2005). KIDSCREEN-52 quality-of-life measure for children and adolescents. Expert Rev Pharmacoecon Outcomes Res.

[62] Goodman R (1997). The Strengths and Difficulties Questionnaire: a research note. J Child Psychol Psychiatry.

[63] Niclasen J, Teasdale TW, Andersen AMN, Skovgaard AM, Elberling H, Obel C (2012). Psychometric Properties of the danish strength and difficulties Questionnaire: the SDQ assessed for more than 70,000 raters in four different cohorts. PLoS ONE.

[64] Møller L (2012). Vejledning til Tekstlæseprøve 1–8 [Guidelines for Reading tests 1–8].

[65] Hansen KF (2014). MG – Matematik Grundlæggende: Diagnosticering Af Grundlæggende Færdigheder I Matematik [Assesment of Basic Skills in Mathematics].

[66] Bulus M, Dong N, Kelcey B, Spybrook J, PowerUpR. Power Analysis Tools for Multilevel Randomized Experiments. 2021 [cited 2023 Mar 23]. Available from: https://CRAN.R-project.org/package=PowerUpR.

[67] Thorpe KE, Zwarenstein M, Oxman AD, Treweek S, Furberg CD, Altman DG (2009). A pragmatic–explanatory continuum indicator summary (PRECIS): a tool to help trial designers. J Clin Epidemiol.

[68] Gustafsson PE, Szczepanski A, Nelson N, Gustafsson PA (2012). Effects of an outdoor education intervention on the mental health of schoolchildren. J Adventure Educ Outdoor Learn.

[69] Fiskum TA, Jacobsen K (2013). Outdoor education gives fewer demands for action regulation and an increased variability of affordances. J Adventure Educ Outdoor Learn.

[70] Moher D, Schulz KF, Altman DG (2001). The CONSORT statement: revised recommendations for improving the quality of reports of parallel-group randomised trials. The Lancet.

[71] Tsigilis N, Theodosiou A. Temporal stability of the intrinsic motivation inventory. Percept Mot Skills. 2003;97(1):271–80. 10.2466/pms.2003.97.1.271 14604050

[72] Gagné M (2003). The role of autonomy support and autonomy orientation in prosocial behavior engagement. Motiv Emot.

[73] Van der Kaap-Deeder J, Soenens B, Ryan RM, Vansteenkiste M. Manual of the basic psychological need satisfaction and frustration scale (BPNSFS). Ghent Univ Belg. 2020. Available from: https://selfdeterminationtheory.org/wp-content/uploads/2022/02/BPNSFS_Complete_2020.pdf.

[74] Bhavsar N, Bartholomew KJ, Quested E, Gucciardi DF, Thøgersen-Ntoumani C, Reeve J (2020). Measuring psychological need states in sport: theoretical considerations and a new measure. Psychol Sport Exerc.

[75] Nissen P, Lemire S, Andersen F (2014). Giving students a voice—A preliminary study of the validity of an ultra brief outcome measure for students: the learning rating scale LRS. Scott J Arts Soc Sci Sci Stud.

[76] Helmke A. Einblicknahme in die Lehr- und Lernsituation (Version 6.1). Die Agentur für Qualitätssicherung, Evaluation und Selbstständigkeit von Schule & Rheinland-Pfalz, Ministeriums für Bildung; 2010. Available from: http://unterrichtsdiagnostik.info/media/files/Link%208_ELL_V6_2.pdf.

[77] Helmke A. Undervisningskvalitet og lærerprofessionalitet. Diagnosticering, evaluering og udvikling af undervisning (org. Unterrichtsqualität und Lehrerprofessionalität. Diagnose, Evaluation und Verbesserung des Unterrichts. Franz Emanuel Weinert gewidmet). 1st ed. Rasmussen J, editor. Dafolo; 2013. Available from: https://www.dafoloforlag.dk/media/4x4pxq2w/7279_helmke-bog_appendiks_til_indblik_i_undervisnings_og_l%C3%A6rin.pdf.

[78] Madsen KL, Aggerholm K (2020). Embodying education - a bildung theoretical approach to movement integration. Nord J Stud Educ Policy.

[79] Madsen KL, Aggerholm K, Jensen JO (2020). Enactive movement integration: results from an action research project. Teach Teach Educ.

[80] Rosseel Y (2012). lavaan: an R Package for Structural equation modeling. J Stat Softw.

[81] Kvale S (1996). An intorduction to qualitative research interviewing.

[82] Christensen U, Krølner R, Nilsson CJ, Lyngbye PW, Hougaard C, Nygaard E (2014). Addressing Social Inequality in Aging by the danish Occupational Social Class Measurement. J Aging Health.

[83] Yuksel HS, Şahin FN, Maksimovic N, Drid P, Bianco A (2020). School-Based intervention programs for preventing obesity and promoting physical activity and fitness: a systematic review. Int J Environ Res Public Health.

[84] Bruhn AL, Hirsch SE, Lloyd JW (2015). Treatment Integrity in school-wide programs: a review of the literature (1993–2012). J Prim Prev.

[85] Durlak JA, DuPre EP (2008). Implementation matters: a review of research on the influence of implementation on program outcomes and the factors affecting implementation. Am J Community Psychol.

[86] Hignett A, White MP, Pahl S, Jenkin R, Froy ML (2018). Evaluation of a surfing programme designed to increase personal well-being and connectedness to the natural environment among ‘at risk’ young people. J Adventure Educ Outdoor Learn.

[87] Fiennes C, Oliver E, Dickson K, Escobar D, Romans A, Oliver S. The Existing Evidence-Base about the Effectiveness of Outdoor Learning. Giving Evidence, UCL Institute of Education, University College London, The Institute for Outdoor Learning; 2015 Nov. Available from: https://givingevidence.files.wordpress.com/2015/03/outdoor-learning-giving-evidence-revised-final-report-nov-2015-etc-v21.pdf.

[88] Patton MQ (1979). Evaluation of program implementation. Eval Stud Rev Annu.

