# Supplementary material for: Efficacy and mechanisms of an education outside the classroom intervention on pupils’ health and education: the MOVEOUT study protocol
Source: BMC Public Health. 2023 Sep 19;23:1825. doi: 10.1186/s12889-023-16618-3 (PMC10510120; doi:10.1186/s12889-023-16618-3)
Supplement: Supplementary file 1 — Additional file 1: Table SA1. The MOVEOUT study video materials. [file 12889_2023_16618_MOESM1_ESM.docx]

Table SA1. The MOVEOUT study video materials

| **Type of material** | **Title of material** | **Language** | **Source** |
| --- | --- | --- | --- |
| Parent information and child consent form guidance | What is EOtC and MOVEOUT? | Danish | youtu.be/kH5kpotYB4M |
| Pupil questionnaire data collection support | How do you feel? | Danish | youtu.be/HdCbWlBCshU  youtu.be/TSD4JUD-zLY |
| Performing randomisation | Randomisation | Danish | youtu.be/Bn50bke7dTo |
