# Supplementary material for: Efficacy and mechanisms of an education outside the classroom intervention on pupils’ health and education: the MOVEOUT study protocol
Source: BMC Public Health. 2023 Sep 19;23:1825. doi: 10.1186/s12889-023-16618-3 (PMC10510120; doi:10.1186/s12889-023-16618-3)
Supplement: Supplementary file 2 — Additional file 2. MOVEOUT SPIRIT 2013 Checklist: Section, item number, and manuscript page number. [file 12889_2023_16618_MOESM2_ESM.doc]

MOVEOUT SPIRIT 2013 Checklist: Section, item number, and manuscript page number

| Section/item | Item No | Addressed on page number |
| --- | --- | --- |
| **Administrative information** | | |
| Title | 1 | 1 |
| Trial registration | 2a | 4 |
|  | 2b | NA |
| Protocol version | 3 | 4 |
| Funding | 4 | 29 |
| Roles and responsibilities | 5a | 1 & 29 |
|  | 5b | 29 |
|  | 5c | 29 |
|  | 5d | NA |
| Introduction |  |  |
| Background and rationale | 6a | 5-9 |
|  | 6b | 9 |
| Objectives | 7 | 9-10 |
| Trial design | 8 | 11-12 |
| Methods: Participants, interventions, and outcomes | | |
| Study setting | 9 | 14 |
| Eligibility criteria | 10 | 14-15 |
| Interventions | 11a | 16-17 (includes reference to full TIDieR checklist) |
|  | 11b | NA |
|  | 11c | 17 & 24 |
|  | 11d | 16 |
| Outcomes | 12 | 18-22 & 24 |
| Participant timeline | 13 | 14-16 |
| Sample size | 14 | 20 |
| Recruitment | 15 | 14-16 |
| **Methods: Assignment of interventions (for controlled trials)** | | |
| Allocation: |  |  |
| Sequence generation | 16a | 17 |
| Allocation concealment mechanism | 16b | NA |
| Implementation | 16c | 17 |
| Blinding (masking) | 17a | 18 |
|  | 17b | 18 |
| **Methods: Data collection, management, and analysis** | | |
| Data collection methods | 18a | 18-22 & 24 |
|  | 18b | NA |
| Data management | 19 | 28 |
| Statistical methods | 20a | 20-23 |
|  | 20b | 21 & 23 |
|  | 20c | 20 |
| **Methods: Monitoring** | | |
| Data monitoring | 21a | NA |
|  | 21b | NA |
| Harms | 22 | NA |
| Auditing | 23 | NA |
| Ethics and dissemination | | |
| Research ethics approval | 24 | 28 |
| Protocol amendments | 25 | NA |
| Consent or assent | 26a | 15 & 28 |
|  | 26b | NA |
| Confidentiality | 27 | Appendix C & D |
| Declaration of interests | 28 | 29 |
| Access to data | 29 | Appendix C & D |
| Ancillary and post-trial care | 30 | NA |
| Dissemination policy | 31a | NA |
|  | 31b | 29-30 |
|  | 31c | 29 |
| Appendices |  |  |
| Informed consent materials | 32 | Appendix C & D |
| Biological specimens | 33 | NA |
