# Supplementary material for: Efficacy and mechanisms of an education outside the classroom intervention on pupils’ health and education: the MOVEOUT study protocol
Source: BMC Public Health. 2023 Sep 19;23:1825. doi: 10.1186/s12889-023-16618-3 (PMC10510120; doi:10.1186/s12889-023-16618-3)
Supplement: Supplementary file 3 — Additional file 3. Information about the project and the processing of data (parents). [file 12889_2023_16618_MOESM3_ESM.pdf]

MOVEOUT

WHAT ARE WE INVESTIGATING?

IIn the research project MOVEOUT, we investigate whether education outside the classroom provides more movement and better school motivation, well-being and learning.

We also examine what it is about education outside the classroom that makes a difference.

WHO IS DOING THE RESEARCH PROJECT?

The project is registered at the Department of Nutrition, Exercise and Sports at University of Copenhagen.

In the project, the University of Copenhagen collaborates with Health Promotion Research at the Steno Diabetes Center Copenhagen, Center for Clinical Research and Prevention, Department of Sports Science and Clinical Biomechanics at the University of Southern Denmark, Teacher Education at VIA University College and Children & Nature – Denmark.

If you have questions about the project, you can contact Glen Nielsen, who is responsible. Send an e-mail to gnielsen@nexs.ku.dk or call tel. +45 35 32 08 69.

Read more at www.moveoutstudy.dk.

KØBENHAVNS UNIVERSITET

DEAR PARENTS

In 2022 and 2023, your child's class will be involved in the Education outside the classroom research project MOVEOUT. The class teachers and the school management have chosen that the class participates in the project.

For you to decide whether your child can participate in the research project, which will take place at the school, please read the following information carefully.

IF YOUR CHILD IS ALLOWED TO PARTICIPATE IN THE RESEARCH PROJECT

- **TICK** and **SIGN** the consent declaration on **pages 7 and 8**.
- **DELIVER** the consent form to your child's teacher at the latest the first day the class is visited by a researcher:

WHAT IS EDUCATION OUTSIDE THE CLASSROOM?

In education outside the classroom, parts of the teaching take place outside the classroom and the school buildings. Typically, in nature, in the city, or in collaboration with cultural and social institutions, or companies.

The goal of education outside the classroom is to make teaching in skills, competencies and concepts more concrete through practical work and direct experience from the outside world.

WHY ARE WE EXAMINING EDUCATION OUTSIDE THE CLASSROOM?

Education outside the classroom seems to allow for movement during the school day – while students get better school motivation and well-being – with the same or slightly better learning.

The new knowledge about education outside the classroom will be used to strengthen good use of Education outside the classroom – knowledge that also benefits your child's class and school..

MOVEOUT is financed by novo nordisk fonden

WHO IS PARTICIPATING IN THE PROJECT?

There are 30 different schools in Denmark that participate in the project with classes from grade 4.-10. All teachers who participate receive a free 2-day course in education outside the classroom and thereby receive the opportunity to obtain a professional qualification in this form of teaching.

WE ARE INVESTIGATING TWO PRIMARY QUESTIONS

- **Does education outside the classroom have a positive effect on students' movement behavior, motivation for schoolwork, well-being and learning?**
- **How is education outside the classroom practiced in a way that strengthens health and learning?**

We examine the two questions by measuring students' movement, motivation for schoolwork, well-being, reading skills and mathematical skills. Movement is measured with an accelerometer. Motivation for schoolwork and well-being is measured with questionnaires that students are introduced to by their teacher. We also obtain data on absence directly from the school register. Learning is measured with tests in reading skills and competencies in mathematics that many teachers use in advance. You, as a parent/guardian, will also receive a questionnaire about the household's education and connection to the labor market, as well as your child's general health.

EDUCATION OUTSIDE THE CLASSROOM ON HALF OF THE SCHOOLS

We randomly select half of the schools in the spring of 2022 to have increased focus on education outside the classroom. Teachers in classes with increased focus on education outside the classroom will attend an education outside the classroom course in April 2022 and will the following school year 2022-2023 do education outside the classroom 5 hours a week or more. The remaining half of teachers in classes without increased focus on education outside the classroom will attend a course in 2023 and teachers are encouraged to focus on education outside the classroom the following school year.

We measure before and after the period in which the classes at half of the participating schools has had an increased focus on education outside the classroom. Students' development is examined by comparing the group of classes that have had an increased focus on education outside the classroom, with the group of the other classes that have had their usual teaching. It provides high quality in research, that we can compare classes from two groups of schools with each other.

HOW IS YOUR CHILD'S MOVEMENT MEASURED?

We measure your child's movement 4 times in 2 years: 2x times before the school year 2022-2023, and twice during the school year 2022-2023.

Each time, your child should wear an accelerometer for 7 days. We will come out to the school and attach the accelerometer to your child's thigh with a patch and pick it up again at school after the 7 days. A female and male researcher/assistant will be present to attach the accelerometer on the corresponding gender.

The accelerometer cannot tell anything about how your child moves or where your child is. The accelerometer is not a GPS. The accelerometers can only tell how much your child is moving.

The accelerometer is very small, thin, and light. It can withstand anything, also hard strokes, sports, swimming, and sauna. We use approved and skin-friendly products to attach the accelerometer to the thigh.

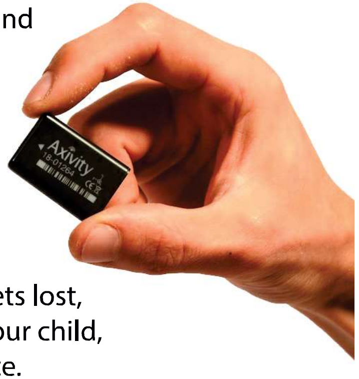

Should there, contrary to expectation, be one motion meter that breaks or gets lost, it is of course, not something you or your child, your child's class or school must replace.

EDUCATION OUTSIDE THE CLASSROOM CLASSES: EXTRA VISIT BY A RESEARCHER

The classes that have increased focus on education outside the classroom will get extra visits from a researcher 4 school days in the school year 2022-2023. The researcher will measure the children's movement on the school day itself, their motivation for schoolwork and their experience of teaching. The researcher will also observe the teaching.

At these extra visits, the students attach the accelerometer themselves in a belt that sits outside their clothes.

WHAT IS IN IT FOR THE CLASS?

In addition to a varied school day and an expected good and educational experience, the class are thanked, with DKK 1,500 to use in the class.

WHEN WILL THE RESULTS OF THE PROJECT BE PUBLISHED?

The project will begin in January 2022. The results are expected to be published before the end of 2025.

WHO GETS TO KNOW THAT MY CHILD IS PARTICIPATING IN THE PROJECT?

The class teacher(s) and researchers on the project know if your child is participating. We make sure that no one else gets this information. The participation is thus fully confidential.

The data we collect about your child and you/parents will not be able to identify your child or you. Read more on the next pages about **what we do with your and your child's data**.

CHILD CERTIFICATE AND COVID-19

In the presence of researchers at the school, requirements are met regarding child certificate and applicable corona guidelines for protection against the spread of infection.

PHOTOS ARE TAKEN TO CONVEY THE RESEARCH

In the classes that have increased focus on education outside the classroom we take photos of the teaching. Photos will in no way be perceived as offensive, humiliating, or offensive. Photos are only used to document how education outside the classroom and the usual teaching is practiced, and to convey the results of the project and the project overall.

Photos may be published:

- In scientific articles, books, reports, and the like.
- In connection with lectures and presentations from the project's sender and partners.
- On media for the project's sender and partners.

CAN MY CHILD BE INJURED BY PARTICIPATING IN THE PROJECT?

No. We do not expect your child to be harmed in connection with the project. All the teaching activities included in the project are reminiscent of the activities your child engages in already when he/she usually goes to school.

The students' teacher(s) attends a 2-day course in education outside the classroom before the project begins. The teacher(s) are responsible for the teaching activities.

There are no needles or procedures. The patches that we use to attach the accelerometer to the thigh is approved, skin-friendly and tested successfully in similar projects.

WHAT DO WE DO WITH YOUR AND YOUR CHILD'S DATA?

PURPOSE OF THIS INFORMATION

We want to make sure that you are properly informed about:

- How your and your child's data will be processed under and after participating in MOVEOUT.
- Who processes your and your child's data.

Therefore, we ask you to read the information on this and the next two pages thoroughly.

THE PROJECT IS ETHICALLY APPROVED

The research project MOVEOUT is ethically approved by the Scientific Ethics Committee at the Faculty of Science, University of Copenhagen.

THE RESEARCH PROJECT IS REGISTERED AT THE UNIVERSITY OF COPENHAGEN

Statutory lists are carried out over the data controllers and data processors and their processing of the personal data included in the research project.

Do you have further questions about how we treat your personal information, you can contact the University of Copenhagen data protection adviser at dpo@adm.ku.dk.

CONFIDENTIAL AND PROTECTED TREATMENT OF YOUR AND YOUR CHILD'S PERSONAL DATA

You and your child's personal data will be treated confidentially from the moment they are collected, until they are deleted or anonymized, or archived in the National Archives after the rules of the Archives Act.

Everyone who processes your and your child's personal data will comply with the duty of confidentiality and every necessary security measure to ensure that your and your child's information does not get accessed by unauthorized persons or be subjected to abuse.

The research project and the processing of your and your child's personal data are registered on The University of Copenhagen's statutory list for the Danish Data Protection Agency. You can read about your rights in connection with our treatment of yours and yours child information in the privacy policy of the University of Copenhagen (www.informationssikkerhed.ku.dk).

The project manager from the University of Copenhagen will work with the research staff from the University of Copenhagen, Steno Diabetes Center Copenhagen, Center for Clinical Research and Prevention, University of Southern Denmark and VIA University College to ensure:

- That your and your child's identity is protected and your and your child's personal data is always processed, stored, and shared in accordance with Danish law data protection, i.e. the Data Protection Regulation (GDPR) and the Danish data protection law.
- That your and your child's identity is protected when the results of the experiment are published.
- That your and your child's personal data are not shared with countries outside the EU/EEA, where data laws may be less stringent (e.g. US).

APPEAL

If you believe that we have not processed your personal data properly, you can complain to The Danish Data Protection Agency, Carl Jakobsensvej 35, 2500 Copenhagen, at dt@datatilsynet.dk. However, you should first contact the University of Copenhagen data protection adviser at dpo@adm.ku.dk.

PARTICIPANT-ID

When your child participates in the research project, your child is assigned a unique participant ID number. This participant ID will be the only thing that identifies you and your child in relation to all your personal data.

The participant ID is also included in a ”key” that connects your and your child’s personal data with your personally identifiable information, such as your or your child’s name and contact information. The ”key” is stored separate from your personal data. The ”key” is stored on a high level of safety and is only dealt with by the researcher and research staff.

- The ”key” will only be used to identify you and your child if this is necessary, such as when we need to contact you in connection with the research project or after the research project has been completed.
- The ”key” can always be accessed by the national or international government officials who have a right to access your and your child’s personal information if they need to verify that the research project has been carried out correctly.
- The ”key” is kept at the University of Copenhagen for as long as it is required by the rules of the research field including the rules on scientific dishonesty and the health science legislation.
- The ”key” and personally identifiable information are stored University of Copenhagen for 5 years after the research project results have been published.

USE OF DATA PROCESSORS

Your and your child’s personal data may be processed by external data processors, e.g. researchers at other universities or professional colleges. External data processors must only process data as instructed by the University of Copenhagen.

When your child participates in the research in the project, your child is assigned a unique participant ID number. The ”key” that connects the participant ID with personally identifiable information will not be disclosed along with your and your child’s personal data when processed by other researchers or data processors who are not part of the project’s research staff from the University of Copenhagen, Steno Diabetes Center Copenhagen, Center for Clinical Research and Prevention, University of Southern Denmark or VIA University College.

There may be use of a data processor outside Denmark, but not countries that can have a less stringent data legislation than in Denmark, e.g. countries outside the EU/EEA.

After treatment, your and your child’s personal data will be returned to Copenhagen University. It may be necessary to be able to document the data quality, that the data processor stores their measurement results for a certain number of years (e.g. 5 years) after the results have been published.

PUBLICATION OF RESULTS

Whatever we find in the project, the results will be published after the end of the project. We publish, for example, the results in scientific journals, reports, book chapters or e.g. as a summary on the University of Copenhagen’s website. Published results do not contain any information that can identify you or your child.

The scientific journals where we try to publish the results can get provided your personal data encrypted with the participant ID. It allows for the journal experts to check the quality of the research without you being identified.

YOU HAVE THE RIGHT TO STOP YOUR CHILDS PARTICIPATION AT ANY TIME DURING THE RESEARCH PROJECT

If you choose to stop your child’s participation in the research project before the project is completed, we will delete the data we have collected about you and your child.

INFORMATION ABOUT YOU AND YOUR CHILD, THAT IS COLLECTED AND STORED

In connection with your child’s participation in the research project, we collect the following information about you and your child, which are stored electronically in a secure database.

INFORMATION	METHOD OF COLLECTION	HOW OFTEN DATA IS COLLECTED	
		ALL CLASSES	CLASSES WITH THE INCREASE FOCUS ON EDUCATION OUTSIDE THE CLASSROOM
How much your child moves in a week	Accelerometer	4x over 7 days	
How much your child moves on a school day	Accelerometer		Up to 4x over 1 day
Your child's academic achievement in reading and math	Tests are conducted by the class teachers	2x	
Your child's general well-being	Questionnaire for the child during school hours	2x	
Your child's general motivation for schoolwork	Questionnaire for the child during school hours	2x	
Your child's motivation during the school day	Questionnaire for the child during school hours		4x
Your child's experience of the teaching environment	Questionnaire for the child during school hours		4x
Your child's and classmates' participation in the teaching	Observation and photos		4x
The class' experience of the teaching environment	Interview in which the child participates with some classmates		1x
UNILOGIN username (used for administering professional tests)	Informed by your child in connection with answering the questionnaire	1x	
Absence from school	Obtained via. school administration	2x	
The child's name, age and possibly diseases and diagnoses	Background questionnaire for parents	1x	
Parents' name, contact information and signature	Declaration of consent	1x	
Parents' education and connection to the labor market	Questionnaire for parents	1x	

REMEMBER THAT YOU MUST CHECK X AND SIGN ON THE NEXT 2 PAGES

GIVE YOUR CONSENT BY TICKING THE BOXES AND SIGN

TICK, SIGN AND HAND IN
TO YOUR CHILDS TEACHER(S) BY THE LATEST

MOVEOUT

CONSENTS ✗ THAT ARE MANDATORY

☐ I am informed about the research project and my child can and would like to participate. I can withdraw my consent at any time by contacting Glen Nielsen, who is in charge of the project, by sending an e-mail to gnielsen@nexs.ku.dk or call tel. +45 35 32 08 69.

☐ My and my child's data will be collected and processed within the purpose of this research project and entered in an evaluation report, in research publications and other publication of the project. When our data is processed, shared with others and published, our names or other directly identifiable information about us are removed and in instead we are coded (pseudonymized) with a number.

☐ I have read how mine and my child's personal data are processed and accepts that our personal data is processed as described in the information material in connection with my children's participation in the research project MOVEOUT.

☐ I understand that it is not essential for my child to participate in the research project, whether I opt in or out above non-mandatory consent to treatments of our personal data, etc.

CONSENTS ✗ THAT ARE NOT MANDATORY

☐ Yes, our data, in pseudonymous form, encoded with my child's participant ID, can following instructions from the University of Copenhagen be shared with other researchers who are not collaborators on the research project. This will be done solely to increase knowledge in the same field, purpose and in the public interest.

Students, for example from the University of Copenhagen, can as part of their education participate in collecting data in the project. Your data can also be shared with students for use in their assignments and reports. These assignments and reports will never include results or information that could identify you or your child.

☐ Yes, our data, in pseudonymous form, encoded with my child's participant ID, can according to instructions from the University of Copenhagen, be shared with students for use in their tasks and reports.

☐ Yes, I would like to be contacted again to participate in similar research projects.

Can photos of your child in teaching situations be published, e.g. on websites, posts on social media, books, and the like?

☐ Yes, photos of my child can be published in connection with the dissemination of the project.

☐ Yes, photos of my child can be published in connection with communication about education outside the classroom from the institutions that collaborate on the project.

☐ I want to be informed about the results of the project.

REMEMBER TO SIGN ON THE NEXT PAGE

SIGNATURE AND CONTACT INFORMATION

MOVE OUT

INFORMATION ABOUT YOUR CHILD

> PLEASE FILL OUT IN BLOCK LETTERS

Name: ____

Birthday (dd.mm.yyyy): _______________ Sex: _____________

School: ____

Class (school year 2021-22) e.g. 7.A:________________________

WHO IS DOING THE RESEARCH PROJECT?

The project is registered at the Department of Nutrition, Exercise and Sports at University of Copenhagen. In the project, the University of Copenhagen collaborates with Health Promotion Research at the Steno Diabetes Center Copenhagen, Center for Clinical Research and Prevention, Department of Sports Science And Clinical Biomechanics at the University of Southern Denmark, Teacher Education at VIA University College and Children & Nature – Denmark.

DATA CONTROLLER

KØBENHAVNS UNIVERSITET

University of Copenhagen
Department of Nutrition, Exercise and Sports
Nørre Allé 51
2200 Copenhagen N

Data about you and your child is kept strictly confidential and consistent with the Personal Data Act and ethical standards for this type of investigation.

CONTACT INFORMATION AND SIGNATURE

> FROM ONE OR BOTH PARENTS

Name: ____

Telephone number: ____________________________________

E-mail: ____

Date: _______________ Signature: __________________________

Name: ____

Telephone number: ____________________________________

E-mail: ____

Date: _______________ Signature: __________________________
