# Supplementary material for: Efficacy and mechanisms of an education outside the classroom intervention on pupils’ health and education: the MOVEOUT study protocol
Source: BMC Public Health. 2023 Sep 19;23:1825. doi: 10.1186/s12889-023-16618-3 (PMC10510120; doi:10.1186/s12889-023-16618-3)
Supplement: Supplementary file 4 — Additional file 4. Information about the project and the processing of data (teachers). [file 12889_2023_16618_MOESM4_ESM.pdf]

MOVE OUT

INFORMATION ABOUT THE PROJECT
AND THE PROCESSING OF YOUR DATA

TEACHERS

novo nordisk fonden
Benefiting people and society

Steno Diabetes Center Copenhagen

KØBENHAVNS UNIVERSITET

Bispebjerg og Frederiksberg Hospital

WHO CONDUCTS THE RESEARCH PROJECT?

University of Copenhagen
Department of Nutrition, Exercise and Sports
Nørre Allé 51
2200 København N.

OVERSEER OF EXPERIMENTS

Glen Nielsen, Lecturer and clusterleader
Mobile: 31 77 12 61
E-mail: gnielsen@nexs.ku.dk

The research project collaborates with two units in the Capital Region: Health promotion research at Steno Diabetes Center Copenhagen and Center for Clinical Research and Prevention at Bispebjerg and Frederiksberg Hospital.

MOVEOUT is financed by Novo Nordisk Foundation.

READ MORE ABOUT THE PROJECT

If you want to know more, then check out the following link: www.moveoutstudy.dk.

HOW TO SIGN UP

READ THE PROJECT INFORMATION

In order for you to consider your participation in the education outside the classroom (EOtC) research project MOVEOUT, and to give your consent, the following pages will provide you with an overview of the project as well as how your data is processed.

You will be informed about what EOtC means in this project, which research activities will take place, and how much time you should set aside for the class(es) you sign up. You can also read about, what the existing research on EOtC shows.

PRINT AND GIVE YOUR CONSENT

After reading the information about the project and the processing of your data, you should:

- **PRINT, TICK OFF, AND SIGN** two declarations of consent: 1) ”consent to participation” og 2) ”consent to the processing of your data”. Both consent declarations have been sent to your email.

- **SCAN** the two declarations of consent, or take a picture of them with your phone, and send them by email to MOVEOUT project leader Mads Bølling (mads.boelling@regionh.dk).

WHAT HAPPENS AFTER REGISTRATION?

1. QUESTIONNAIRE

When we have received your consent, we will send you a link by email to a questionnaire, where we will ask some questions in regards to your experience with EOtC.

2. FIRST VISIT TO THE SCHOOL

During the winter of 2022 you will be contacted by a research employee. Together, you will plan two visits where a research employee can come to the school and add motion detectors to the participants in your class(es).

About 14 days before the first visit, you will receive an email with parent-information as well as a consent form for the parents. This should be printed and given to the pupils to be brought home. During the research employee’s first visit the consent forms should be collected.

3. RESERVE TIME FOR ACADEMIC TESTS

In March 2022, you will receive information concerning how to conduct the academic tests in reading comprehension and maths, Hogrefe SL and MG, in your participating class(es).

4. BE READY FOR ’MAYBE’ ATTENDING A EOTC-SEMINAR IN APRIL 28-29. 2022

On April 1st, we will draw lots between schools on whether they will be starting or have to wait to be offered a two day seminar. All participating teachers will be informed if they are starting or waiting to be offered the seminar.

If you are a teacher in one of the schools which will be offert the seminar first, you will receive the seminar material 14 days before. This includes material which requires about 2 hour preparation time.

In conjunction with drawing lots, all participating teachers will receive more information about the research activities during the school year of 2022-2023.

WHAT IS MOVEOUT?

AN EDUCATION OUTSIDE THE CLASSROOM RESEARCH PROJECT

MOVEOUT is a research project. The project researches the effect of a welltested initiative of a regularly use of EOtC, ie 'Udeskole'. Udeskole consists of didactic and pedagogic work methods, in which education, over a longer period of time, takes place outside of the classroom and the school buildings, in places where education normally isn't scheduled to take place.

RESEARCH QUESTION

In MOVEOUT two overarching research questions are examined:

- Does 'Udeskole' have a positive impact on movement behaviour, motivation for school work, well-being, and academic leraning?
- How is 'Udeskole' best practiced?

RECOGNITION FOR HELP WITH THE RESEARCH PROJECT

As thanks for the help and assistance with the project, participants will receive a giftcard:

- **Teachers** - 1.500 DKK. (taxable).
- **Classes** - 1.500 DKK. for the class (not taxable).

SEMINAR AND ONE YEAR WITH UDESKOLE: STARTING OR WAITING

TEACHERS AND PUPILS

Participants in MOVEOUT are teachers and their pupils in public schools in 4th to 10th grade. Participating teachers in MOVEOUT, receive a free 2-day seminar in EOtC and get the opportunity for a professional upskilling in didactic and pedagogic work methods.

The teachers agree that:

- Lots will be drawn between schools **starting** the seminar on April 28 and 29th 2022, utilising EOtC regularly in the school year 2022-2023 with their participating class(es), 5x 60 minutes a week, split between 1-2 days. The 5x 60 minutes include transportation and breaks as well as preparation time and evaluation on the day itself, which can take place back in the classroom.
- That the participating teachers that are **waiting** to start the seminar, until April 2023, contintue their education as usual in the school year of 2022-2023 with their participating class(es).
- That all teachers set time aside in 2022 and 2023 for research activities (read more in the section on research activities on page 8).
- To convey information to parents and support that the pupils are participating

THE ROLE OF THE PARENTS

Parents answer a questionnaire about their own education and employment situation as well as any potential illness- or diagnosis conditions etc. and consent to:

- That their children can participate in research activities, and that data on their absence potentially can be collected.
- That there potentially can be taken pictures of their children, in connection to observations of the teaching activities, which can be used to document what takes places during teaching activities and for project dissemination.

WHAT IS EOTC IN MOVEOUT?

REGULAR TEACHING ACTIVITIES OUTSIDE THE CLASSROOM AND THE SCHOOL BUILDINGS

Regularly use of EOtC, ie 'Udeskole', consists of didactic and pedagogic work methods, in which education, over a longer period of time, takes place outside of the classroom and the school buildings, in places where education normally isn't scheduled to take place.

EOtC is usually practiced in nature, but also in conjunction with culture- and social institutions, with the intention of making teaching activities in skills, competencies, and concepts more concrete – via a practical and experience-based approach.

WHAT DOES EOTC MEAN FOR TEACHING ACTIVITIES? [1]

- That the aqcuisition of knowledge, skills, and opinions are created through experiences, actions, dialogue and reflection.
- That teaching activities can take place in the surrounding area of the school, in nature, social life, and cultural institutions.
- That the school subjects can be activated in integrated teaching activities, where activities both inside and outside have close similarity.

[1] UdeskoleNet, https://www.skoven-i-skolen.dk/content/udeskole-i-danmark-en-definition

EXTENT OF EOTC IN MOVEOUT

To make it possible to investigate the importance of EOtC, it is important that the teaching in the classes that start practising udeskole in the school year 2022-2023 has a certain scope of character:

- On average 5x 60 minutes per week, spread over 1-2 school days throughout the 2022-2023 school year.
- The 5x 60 minutes includes possible transportation tine in relation to EOtC-sessions, and preparation and evaluation on the day of EOtC-sessions itself.
- Each teaching session with udeskole must have a duration of 45 minutes or more.
- Teaching is delimited by Common Objectives (Fælles Mål) for Danish schools in individual and interdisciplinary subjects.

WORK METHODS IN EOTC

- EOtC is characterised by collaborative, action-oriented, experience- and investigation-based and thematic learning processes.
- EOtC aims to promote students' learning and well-being trhough practical activities and use of body and senses in authentic situations - through interaction between actions and thoughts.

EXAMPLES OF EOTC

PRACTICE THAT SHOULD NOT BE DEFINED AS EOTC

EXAMPLE 1

General physical education, which takes place on the sports field, e.g. in athletics or ball games,

- because the teaching that takes place where the teaching is normally scheduled to take place.

EXAMPLE 2

If it is movement activities without a direct academic learning goal, e.g. 'brain breaks' or

- 'brain breaks' or
- times reserved exclusively for games or play.

Movement activities can, however, be included during a lesson that includes EOtC.

ACTIVITIES THAT CAN BE INCLUDED IN A TEACHING SESSION WITH EOTC

EXAMPLE 1

Maths lessons that take place on the sports field because it is outside the classroom where the lessons are normally scheduled to take place.

EXAMPLE 2

In connection with a teaching session with EOtC, there will often be a short introduction in the classroom before departure and possibly a short summary in the classroom.

- Introduction and evaluation should be considered as part of the EOtC activities.

EXAMPLE 3

If the pupils move outside the school buildings during teaching with an academic aim, where the content of EOtC is an integrated part of the academic teaching. This could be, for example,

- that the pupils must follow a planetary path, where the academic content is an understanding of the mutual distance betweeen the planets and the distance to the sun,
- that pupils work with ethics and environmental behaviour by collecting waste,
- that students solve language-teaching related tasks around the school grounds.

EXAMPLE 4

All teaching activities with a professional aim that take place in whole or in part time museums, cemeteries, in parks, forests, beaches, companies, and the like.

EXAMPLE 5

On school camp days, EOtC is only considered the time spent with teaching activities i.e. both direct teaching, feedback, and activities with an educational focus.

2-DAY SEMINAR IN UDESKOLE

TIME AND PLACE

The seminar will take place at Kattinge Værk in Roskilde, both days betwene 8:30 and 14:30.

The two seminar days are supplemented by two online course sessions of one hour duration each.

Teachers must be able to set aside a total of two hours for preparation for the course.

For **schools, that start** with 'udeskole' and are offerede the seminar, the seminar will be held on Thursday the 28. of april and friday the 29. of April 2022.

For schools that are waiting, dates will be announced in autumn 2022.

TARGET GROUP

The seminar is targeted all teachers, no matter the experience with practising regularly use of EOtC.

PURPOSE AND CONTENT

The purpose of the seminar is to equip teachers to practice Udeskole targeted 4.-10. graders, in one or more subjects as well as interdisciplinarily.

The seminar will, among other things, focus on planning teaching activities, organising the teacing, timetable planning as well as tips and tricks to get started.

At the seminar, there will be time to reflect on one's own EOtC practices and time to start planning Udeskole for the school year 2022-2023.

WORK METHODS

The seminar includes illustrative examples of 'udeskole' in e.g. the major subjects and other subjects where Udeskole is most often practised.

Practical teaching and presentations from experienced Udeskole-teachers, as well as plenary and group discussions will be part of the work methods.

FINANCE

The seminar is free for all participating teachers

The project's financial framework enables up to 2 teachers from each of the 1-3 participating classes in 30 schools to be offered the seminar and can be compensated with DKK 270/h. for up to 14 working hours.

Schools are compensated financially for the working time the teachers use when they participate in the seminar and preparation.

RESEARCH ACTIVITIES

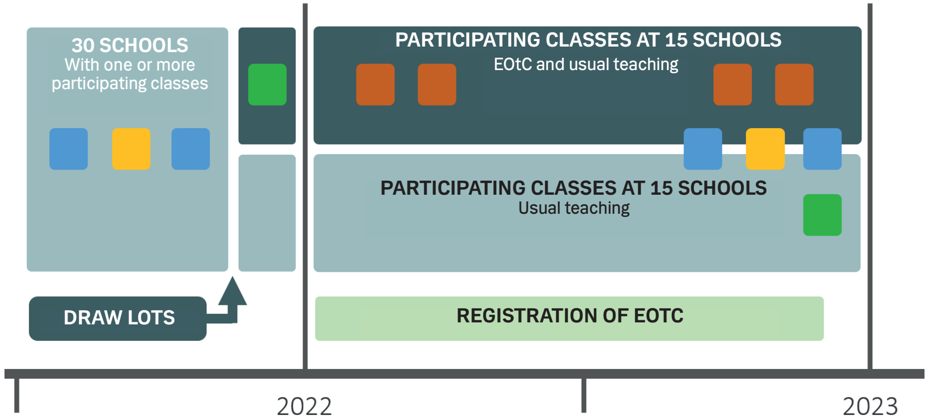

REGISTRATION OF EOTC

THE SCHOOL YEAR 2022-2023

Teachers, both those starting and waiting, record each week whether their registered class(es) have been taught using EOtC or not.

- Registration takes place with a questionnaire that takes 2 to 10 minutes, depending on whether the class has had EOtC or not. The questionnaire is sent by SMS.

CHILD CERTIFICATE AND COVID-19

When research staff are present at school, requirements for child certificates and current Covid-19 guidelines for protection against the spread of infection are complied with.

MEASURING STUDENT MOVEMENTS

FEBRUARY-APRIL 2022 AND 2023

- The pupils wear the movement trackers for 7 days, and the researchers are given a copy of the students' school timetable. The procedure takes 1 lesson and is carried out four times as expected or in either feb./mar. 2022, apr./may 2022, feb./mar. 2023 og apr./may 2023.

MOTIVATION, ACADEMIC TESTS AND ABSENCE AS WELL AS TEACHER EXPERIENCES

APRIL 2022 AND 2023

- Teachers conduct professional tests (Hogrefe SL and MG) (2 lessions).
- Pupils answer a questionnaire, i.a. about their school motivation (1 lesson)
- Teachers answer a questionnaire about their own teaching practice and experience, i.a. with 'udeskole' (expected 30 minutes).
- Data on absences is obtained from the school

OBSERVATION OF EOTC-TEACHING

SEPTEMBER 2022 TO MAY 2023

1-2 research staff observe 2 days of udeskole and 2 other school days in each class (applies only to classes starting, ie. EOtC-classes).

- For each observation, pupils' movement is measured, and pupils answer a short questionnaire about motivation for schoolwork and the learning environment. Photos are taken to document the teaching. Teachers must alocate 1 lesson for research activities the first of the four observation days.
- The teacher participates in an interview (1 hour).

TWO-DAY SEMINAR IN EOTC

28-29TH APRIL 2022 OR TWO DAYS IN APRIL 2023

WHAT EXISTING EOTC RESEARCH SHOWS

GOOD FOR HEALTH, WELLBEING, AND LEARNING

The existing research on reguarly use of EOtC, mainly from the Danish research project TEACHOUT, shows a positive significance of EOtC at the intermediate level pupils' health, wellbeing and learning.

Overall, there is still a great need for more reliable knowledge about EOtC.

POSITIVE EFFECT ON SCHOOL MOTIVATION AND SOCIAL WELLBEING

Reguarly use of EOtC seems to have a positive effect on both school motivation and social wellbeing in th class communities, without seeming to compromise learning.

POSITIVE EFFECT ON READING SKILLS

Although no measurable effect on math skills has been shown, reading skills appear to be strengthened.

ASSOCIATION WITH MORE PHYSICAL ACTIVITY

In general, EOtC is positively associated with physical activity. Both girls and boys have a lower proportion of sedentary behavior on school days with EOtC activities, and separately for boys the proportion of high physical activity is greater on a weekly basis.

EOtC in nature and green areas seems to provide more physical activity, compared to EOtC that does not take place in nature and green areas.

In addition to offering an obvious opportunity for active transport, students move naturally as part of the EOtC learning activities.

EOTC: A GOOD EXPERIENCE

Pupils in Denmark are mostly happy with EOtC. This way of working in school seems to give them an experience of greater variety in teaching.

Teachers see considerable potential in including EOtC as part of the teaching.

DEVELOPMENT OF EOTC REQUIRES SUPPORT

In order for the potential of EOtC to be realized – ie. to strengthen pupils' academic and well-being gains, and movement – there is a need to support the implementation of EOtC locally at the individual school.

Courses, knowledge sharing and inspiration for how teachers can work in a targeted way with EOtC can support the development of EOtC locally at the individual school.

ON THE FOLLOWING PAGES YOU CAN READ ABOUT OUR PROCESSING OF YOUR DATA

YOUR DATA?

The purpose of this information is that we will make sure that you are properly informed about:

- How your data will be processed during and after participation in MOVEOUT.
- Who processes your data.

We therefore ask you to read the following information carefully.

WHO IS BEHIND THE PROJECT?

In the research project, the Department of Nutrition, Exercise and Sports at the University of Copenhagen collaborates with two units in the Capital Region: Health promotion research at the Steno Diabetes Center Copenhagen and the Center for Clinical Research and Prevention at Bispebjerg and Frederiksberg Hospital.

CONFIDENTIAL AND PROTECTED TREATMENT OF YOUR PERSONAL DATA

Your personal data will be treated confidentially from the moment it is collected from you until it is deleted or anonymised, or archived in the National Archives in accordance with the regulations in the Archives Act.

Everyone who processes your personal data will comply with the duty of confidentiality and all necessary security measures to ensure that your information cannot be accessed by unauthorized persons or exposed to misuse.

The research project and the processing of your personal data are registered on the University of Copenhagen's statutory register to the Data Protection Authority.

You can read about your rights in connection with our processing of your information in the privacy policy for the University of Copenhagen (https://informationssikkerhed.ku.dk/).

The experiment manager from the University of Copenhagen, together with the research staff from the University of Copenhagen and the Capital Region, will ensure:

- That your identity is protected and your personal data is always processed, stored and shared in accordance with Danish legislation on data protection, i.e. the General Data Protection Regulation (GDPR) and the Danish Data Protection Act.
- That your identity is protected when the results of the trial are published.
- That your personal data is not shared with countries outside the EU/EEA, where data legislation may be less strict (e.g. USA).

USE OF DATA PROCESSORS

Your personal data may be processed by external data processors, e.g. external researchers at other universities or university colleges.

External data processors may only process data according to instructions from the University of Copenhagen.

When you participate in the research project, you will be assigned a unique participant ID number. The ”key” that connects the participant ID with personally identifiable information will not be handed out together with your personal data when it is processed by other researchers or data processors who are not part of the project’s research staff from the University of Copenhagen or the Capital Region.

There may be use of data processors outside of Denmark, but not countries that may have less strict data legislation than in Denmark, e.g. countries outside the EU/EEA.

After the end of processing, your personal data will be returned to the University of Copenhagen. In order to be able to document the data quality, it may be necessary for the data processor to store their measurement results for a certain number of years (e.g. 5 years) after the results have been published.

PARTICIPANT-ID

When you participate in the research project, you will be assigned a unique **participant-ID-number**. This participant ID will then be the only thing that identifies you in relation to all your personal data.

The participant ID is also part of a ”key” that connects your personal data with your personally identifiable information, such as your name and contact information. The ”key” is always stored separately from your personal data. The ”key” is kept at a high level of security and is only processed by the research manager and research staff.

- The ”key” will not be handed out together with your personal data when it is processed by other researchers or data processors who are not part of the project’s research staff from the University of Copenhagen or the Capital Region.
- The ”key” will only be used to identify you if this is necessary, such as when we need to contact you in connection with the research project or after the research project has been completed.
- The ”key” can always be accessed by the national or international authorities who are entitled to access your personal data if they need to check that the research project has been carried out correctly.
- The ”key” is kept at the University of Copenhagen for as long as is required by the rules in the field of research, including the rules on scientific misconduct and the health science legislation.
- The ”key” and personally identifiable information are stored by the University of Copenhagen for 5 years after the results of the research project have been published.

ADDITIONAL INFORMATION

YOU HAVE THE RIGHT TO STOP YOUR PARTICIPATION IN THE RESEARCH PROJECT

If you choose to stop your participation before the research project is completed, the data that we have collected can still be included in the overall data analysis if this is necessary for the quality of the trial.

The personal data we already have from you will therefore only continue to be processed if it is factual and important for the research project.

PUBLICATION OF RESULTS

Regardless of what we find out in the research project, the results will be published some time after the end of the project.

We publish e.g. the results in scientific journals, reports, book chapters or e.g. as a summary on the University of Copenhagen's website. Published results do not contain any information that can identify you.

The scientific journals in which we try to publish the results of the trial can be given your personal data coded with the participant ID. This enables the journal's experts to check the quality of the research without you being identified.

MOVEOUT HAS BEEN ETHICALLY APPROVED

MOVEOUT is ethically approved by the Scientific Ethics Committee at The Faculty of Science at University of Copenhagen.

INFORMATION ABOUT YOU THAT IS COLLECTED AND STORED

In connection with your participation in the research project, we collect the following information about you, which is stored electronically in a database.

INFORMATION	COLLECTION METHOD	HOW OFTEN DATA IS COLLECTED
Name, age, and gender	Questionnaire	1x
Contact information (mobile, e-mail)	Consent form	1x
Contact info nearest leader	Recruitment	1x
Education and educational experience	Questionnaire	1x
Your evaluation of the course	Questionnaire	1x
Your experince using udeskole	Questionnaire	1x
Reflections on own practice	Interview	1x (possibly)
Your teaching, in practice	Observation, photo	4x [1]
Your participation in the course	Photos	1x

[1] Applicable to classes start using EOtC, ie. EOtC-classes.

THE RESEARCH PROJECT IS REGISTERED AT THE UNIVERSITY OF COPENHAGEN

Statutory records are kept of the data controller and data processors and their processing of the personal data included in the experiment. If you have further questions about how we process your personal data, you can contact the University of Copenhagen's data protection advisor at the e-mail address dpo@adm.ku.dk.

APPEAL OPTION

If you believe that we have not processed your personal data properly, you can complain to the Data Protection Authority, Carl Jakobsensvej 35, 2500 Copenhagen, at the e-mail address dt@datatilsynet.dk. However, you should first contact the University of Copenhagen's data protection advisor.

MOVEOUT
